# Attrition from Web-Based Cognitive Testing: A Repeated Measures Comparison of Gamification Techniques

**DOI:** 10.2196/jmir.8473

**Published:** 2017-11-22

**Authors:** Jim Lumsden, Andy Skinner, David Coyle, Natalia Lawrence, Marcus Munafo

**Affiliations:** ^1^ UK Centre for Tobacco and Alcohol Studies School of Experimental Psychology University of Bristol Bristol United Kingdom; ^2^ The MRC Integrative Epidemiology Unit University of Bristol Bristol United Kingdom; ^3^ School of Computer Science University College Dublin Dublin Ireland; ^4^ School of Psychology College of Life and Environmental Sciences University of Exeter Exeter United Kingdom

**Keywords:** behavioral research/methods, games, experimental, computers, cognition, Internet, play and playthings/psychology, boredom, task performance and analysis, executive function, inhibition (psychology)

## Abstract

**Background:**

The prospect of assessing cognition longitudinally and remotely is attractive to researchers, health practitioners, and pharmaceutical companies alike. However, such repeated testing regimes place a considerable burden on participants, and with cognitive tasks typically being regarded as effortful and unengaging, these studies may experience high levels of participant attrition. One potential solution is to gamify these tasks to make them more engaging: increasing participant willingness to take part and reducing attrition. However, such an approach must balance task validity with the introduction of entertaining gamelike elements.

**Objective:**

This study aims to investigate the effects of gamelike features on participant attrition using a between-subjects, longitudinal Web-based testing study.

**Methods:**

We used three variants of a common cognitive task, the Stop Signal Task (SST), with a single gamelike feature in each: one variant where points were rewarded for performing optimally; another where the task was given a graphical theme; and a third variant, which was a standard SST and served as a control condition. Participants completed four compulsory test sessions over 4 consecutive days before entering a 6-day voluntary testing period where they faced a daily decision to either drop out or continue taking part. Participants were paid for each session they completed.

**Results:**

A total of 482 participants signed up to take part in the study, with 265 completing the requisite four consecutive test sessions. No evidence of an effect of gamification on attrition was observed. A log-rank test showed no evidence of a difference in dropout rates between task variants (χ^2^_2_=3.0, *P*=.22), and a one-way analysis of variance of the mean number of sessions completed per participant in each variant also showed no evidence of a difference (F_2,262_=1.534, *P*=.21, partial η^2^=0.012).

**Conclusions:**

Our findings raise doubts about the ability of gamification to reduce attrition from longitudinal cognitive testing studies.

## Introduction

The prospect of assessing cognition remotely and longitudinally is attractive to researchers, health practitioners, and pharmaceutical companies alike. However, traditionally, assessments of cognitive functioning are performed in a laboratory or clinical setting, making multiple testing sessions expensive and a burden to both researchers and participants. At the time of writing, the use of Internet-based platforms for crowdsourcing participants, such as Amazon MTurk [1] and Prolific Academic [2], combined with the growing number of platforms for delivering Web-based cognitive assessments, such as Testable [3] and Gorilla [4], has newly given researchers the ability to gather data on large numbers of people within very short time spans [5-8]. These new technologies have allowed psychological experiments and interventions to be delivered via the Web easily and inexpensively [9-11].

However, one issue for Web-based studies (and particularly longitudinal studies) is that they must compete against the wealth of entertainment and distraction available on the Internet to attract and retain their participants. This is made more difficult by the fact that dropping out of a Web-based study is easier than doing so in the laboratory, that is, a participant needs to only close their browser window [12]. Many authors have reported difficulties sustaining participant numbers for the duration of their Internet-based studies [13,14], and reviews of adherence to intervention trials have documented dropout rates of around 50% [15,16], considerably higher than in laboratory studies where dropout rates are around 13% [17]. The gradual reduction in the number of participants who continue to provide study data over time is known as attrition [16,18]. High levels of attrition may cause studies to suffer from smaller than intended sample sizes, incomplete datasets, wasted participant compensation, and potentially biased results [19-21].

Attrition is often characterized as a “lack of participant engagement” [22,23], but the definition of *engagement* is unclear. One potential definition conceptualizes engagement in a twofold sense [24], both referring to participants’ subjective experience of taking part in a study (ie, their enjoyment of the procedure) and participants’ behavior when interacting with the study (ie, how often they return to the study website, or how quickly they drop out). Under this definition, attrition is a subcomponent of engagement: an objective behavioral measure that could be assumed to relate to the concept of engagement as a whole.

Gamification has been heralded as a potential mechanism for increasing participant engagement with Web-based studies and interventions [25-27]. The premise is that by adding gamelike features (points, graphics, levels, competition, etc) to an otherwise mundane task, we might be able to create a more enjoyable and compelling experience for the user [28-30]. By utilizing games’ ability to engage individuals, it may be possible to make the testing experience less burdensome, thereby reducing attrition. In previous studies, self-report questionnaires of participants’ enjoyment have found that gamelike experiments are typically rated as more enjoyable than their nongamelike counterparts [25,31-35]. There are also some examples of gamification increasing *objective* measures of engagement, such as number of optional trials completed [34] or the number of optional testing blocks chosen [36].

Two systematic reviews looked at the effect of gamification on engagement with “online programs” (mostly e-learning) [37] and Web-based mental health interventions [38]. Drawing on the data from 15 studies comparing engagement with gamified programs with nongamified programs, Looyestyn et al [37] found medium to large effects of gamification on objective measures of engagement such as time spent using the program, number of website visits, and volume of contributions. In contrast, Brown et al [38] assessed the impact of gamification on adherence to 61 Internet-based mental health interventions and found that not only was gamification applied fairly lightly (most studies used only one gamelike element) but there was also little evidence for its efficacy. These conflicting findings could be the result of the reviews’ different scopes, the lack of studies in Brown’s review which specifically assessed the impact of gamification on adherence, or the very minimal gamification found to have been applied in the reviewed mental health interventions.

The reluctance of researchers to liberally apply gamification to precisely designed mental health interventions is understandable; any small change might impact the intervention’s efficacy. Within our own field of gamified cognitive assessment, efforts to increase participant engagement and reduce attrition must be implemented carefully to avoid introducing additional cognitive load and affecting the cognitive constructs under test, thus invalidating the task. Although some studies have reported a positive effect of game mechanics on participant performance [39-41], others have found evidence that gamelike tests *do not* improve performance, and may in fact worsen it [31-33,42-44]. For example, Katz et al [42] found that adding a point scoring system to a working memory training task negatively impacted the task’s ability to train cognition. These contrasted findings are likely due to the diverse range of cognitive tasks being used and the variety of gamification approaches applied to them, thus highlighting the need for research, which systematically manipulates gamification techniques within a single type of task [25].

We previously conducted a study exploring the impact of two simple game mechanics (points and theme) on the data collected by, and subjective participant ratings of, a response inhibition task [43]. The points variant rewarded participants with points in accordance with their performance on the task, whereas the theme variant utilized a variety of narratively themed stimuli and task backgrounds. A nongame variant was included as a control condition. This was comparable with a clinical version of the task, with some minor graphical changes to ensure suitability for online use. We found that points were rated highest of the three variants on a subjective questionnaire of enjoyment and engagement and did not negatively affect participant performance on the test. However, we found that the narratively themed task was less liked and negatively affected participant performance. We also observed ceiling effects on participant accuracy in all three task variants because of the ease of the response inhibition task we used.

In this study, we aimed to investigate whether simple gamification could reduce participant attrition from a Web-based longitudinal cognitive testing study. Building on our previous study, we used three variants of a response inhibition task, but we switched to using the Stop Signal Task (SST) to increase task difficulty and avoid ceiling effects. We used the same gamelike features (nongame, points, and theme) as in the previous study. We aimed to assess the effect of gamification on attrition using a longitudinal design whereby participants signed up to four compulsory test sessions over 4 consecutive days before entering a 6-day voluntary period where they could continue to take part once per day if they desired. Participants were told that they would receive £4 for completing all compulsory sessions and an additional 50p for each optional session they completed.

We hypothesized that the nongame variant would have the highest attrition rate, losing participants quickly once the fourth session was complete. We expected the points variant initially to maintain high numbers before falling rapidly around day 6 and 7. Finally, we expected the theme variant to lose participants steadily at first before stabilizing to a low attrition rate, eventually retaining a higher number of participants than either the nongame or points variants. For more information on why we predicted these hypotheses, see Multimedia Appendix 1.

## Methods

### Design and Overview

We used a between-subjects repeated measures experimental design that took place online over 4 to 10 days. The independent variable was SST variant (nongame, points, and theme). The dependent variables of interest were participant attrition, scores on a questionnaire of enjoyment and engagement, two pilot objective measures of engagement, and stop signal reaction times (SSRTs). We preregistered the study on the Open Science Framework [45].

### Participants and Procedure

Participants were recruited from the user base of Prolific Academic [2], which handles the process of checking inclusion criteria, displaying study information, and participant reimbursement. We required participants to be older than 18 years and to have English as a first language but had no further criteria. Once registered, participants were directed to the *Mindgames platform* where they entered their prolific ID and received a unique link, which they used to access the study thereafter. They were then randomly assigned to a single task variant for the duration of the study and completed a Web-based consent form before the testing commenced.

Participants were required to complete one 10-min session per day for the first 4 days of the study to receive £4 as compensation for their time. If participants dropped out of the study before completing four sessions and did not contact us with a reason (technical difficulties, etc), then they did not receive any compensation. This was made clear on the information sheet, which participants read before they signed up to the study, and on the study website itself. For the first four sessions, participants were sent daily reminders via the Prolific Academic messaging system. On the fourth day, participants were informed that there would be no more reminders, and that they were free to either drop out or continue to take part in the study each day thereafter for up to 6 days, with each additional session earning them 50p, for a total of between £4 and £7.

The appropriate compensation for the optional sessions was determined by way of a pilot study using the nongame variant only. We randomly allocated participants to one of the three levels of compensation, that is, 50p, £1, or £2 per optional session completed (the base compensation was still £4) and found that the average number of sessions completed per participant was 7.1, 8.4, and 9.4, respectively. Given that we anticipated the nongame variant to be the least motivating of the three variants, that we wanted to avoid ceiling effects, and that we wanted to minimize the motivational influence of the compensation, we opted for a reward of 50p per optional session.

Ethics approval was obtained from the Faculty of Science Research Ethics Committee at the University of Bristol (40361), and the study was conducted according to the revised Declaration of Helsinki [46].

### Materials

#### The Mindgames Platform

**Figure 1:**
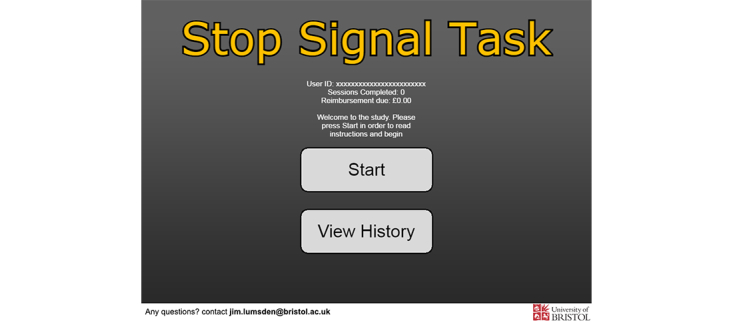
Menu screen of the nongame task variant.

**Figure 2:**
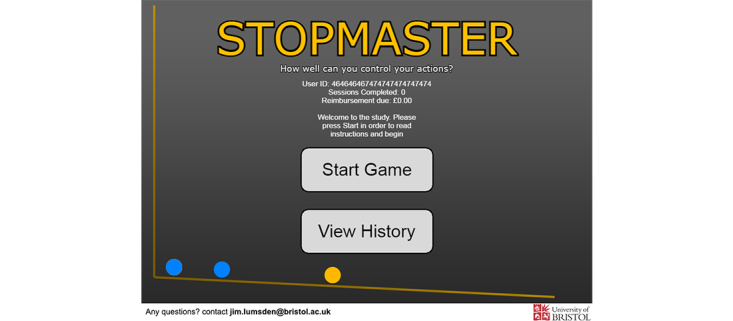
Menu screen of the points task variant.

**Figure 3:**
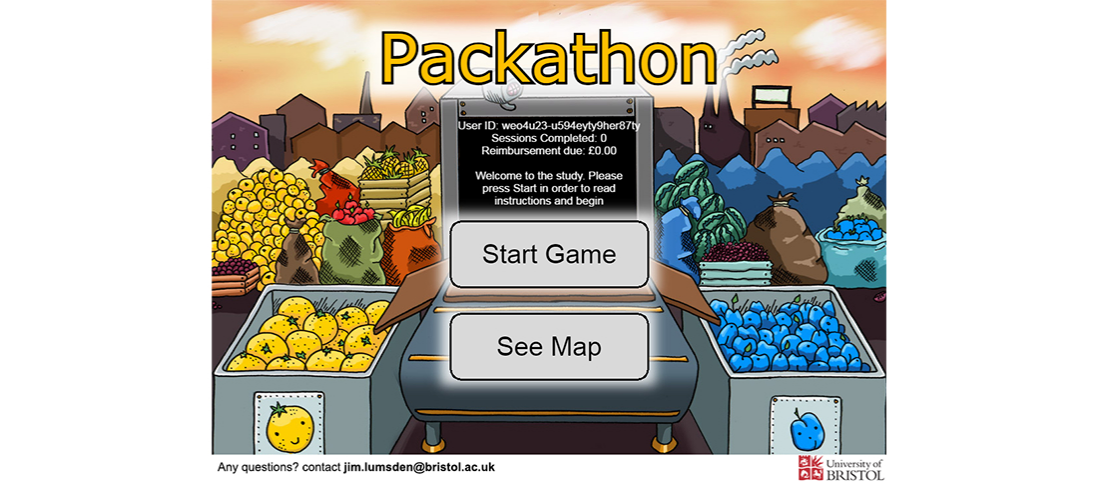
Menu screen of the theme task variant.

Aside from participant recruitment, daily reminders, and reimbursement, all other elements of the study were hosted on a custom website [47]. The website was a single-page Web app written in JavaScript, with a JSON-based Firebase database [48] and PixiJS [49] as the 2D renderer. The site opened to a main menu screen from which the participant could view the number of sessions they had completed and the amount of money they had earned so far (Figures 1-3). Participants had access to a *history* screen, which allowed them to view their previous progress and monitor their results over time. Clicking the start button displayed a series of instruction screens, followed by the SST task and a short questionnaire. The session ended on the history screen, and the main menu’s *start* button became inactive until midnight that night. Each session took approximately 10 min to complete. On the first day of taking part, participants also completed a short demographic questionnaire, which collected data on age, sex, ethnicity, level of education, and the number of hours spent playing video games each week.

#### Stop Signal Task: Nongame Variant

The SST measures response inhibition [50,51], a key feature of executive control [52]. It tests the participant’s “action restraint” by presenting a series of stimuli to which the participant must respond as quickly as possible but are occasionally required to withhold a response. These *stop trials* are indicated by a visual warning that presented a brief delay after stimulus presentation. The primary outcome measure of the SST is the SSRT, which is the number of milliseconds of warning a participant needs for them to be able to successfully inhibit their planned response [52].

In this study, we decided to use the SST as opposed to the Go-NoGo task from our previous study [43]. We did this because we found many participants to be performing at ceiling in the Go-NoGo task, which limited our ability to detect differences between the task variants. The SST is more challenging than the Go-NoGo task because it dynamically adjusts the task’s difficulty to match the inhibitory control of the user, therefore reducing the likelihood of a participant performing at ceiling.

**Figure 4:**
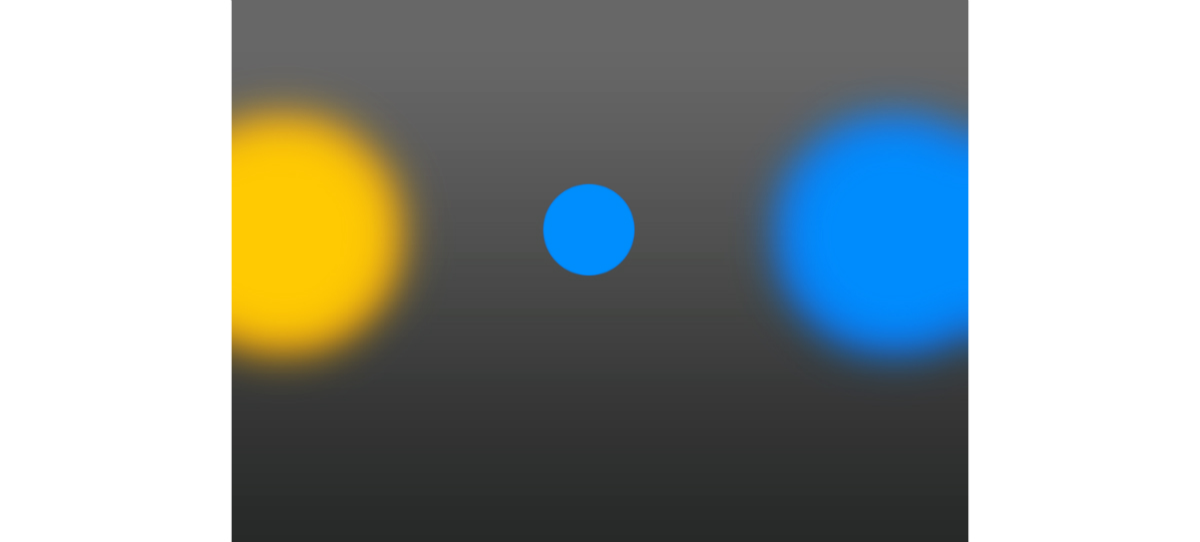
Screenshot of the nongame stop signal task.

We based our SST on the widely used CANTAB SST [53,54] albeit with a visual rather than auditory stop signal and some graphical upgrades to make the task more suitable for the Web. Each trial began with a fixation cross that was displayed in the middle of the screen, with two colored zones on the left and right of the fixation cross (Figure 4). After 500 ms, a colored circle appeared over the fixation cross and participants had to respond as rapidly as possible by pressing either the left or right arrow key to indicate which colored zone matched the color of the circle (Multimedia Appendix 2). In 25% of trials, white brackets appeared around the circle after it was shown: when this occurred, the subject had to withhold their response and wait until the next trial began (each trial was displayed for 900 ms). If the participant responded before the stop signal was displayed, then the trial was recorded as failed, but white brackets were not displayed. Between each trial, there was a random intertrial interval ranging from 500 to 1000 ms. The delay between the circle onset and the bracket onset is called the stop signal delay (SSD), and was varied according to a four-staircase tracking algorithm, designed to sample across the *inhibition-probability by SSD* space (see Multimedia Appendix 1) [55,56]. The task consisted of five blocks of 48 trials each, with a 10s break between each block. If the participant minimized the browser window or changed tabs, then the task was paused (because of the default way in which timers in JavaScript operate). However, if the browser window was not in focus but was still visible (eg, on a second monitor), then the task was not paused.

**Figure 5:**
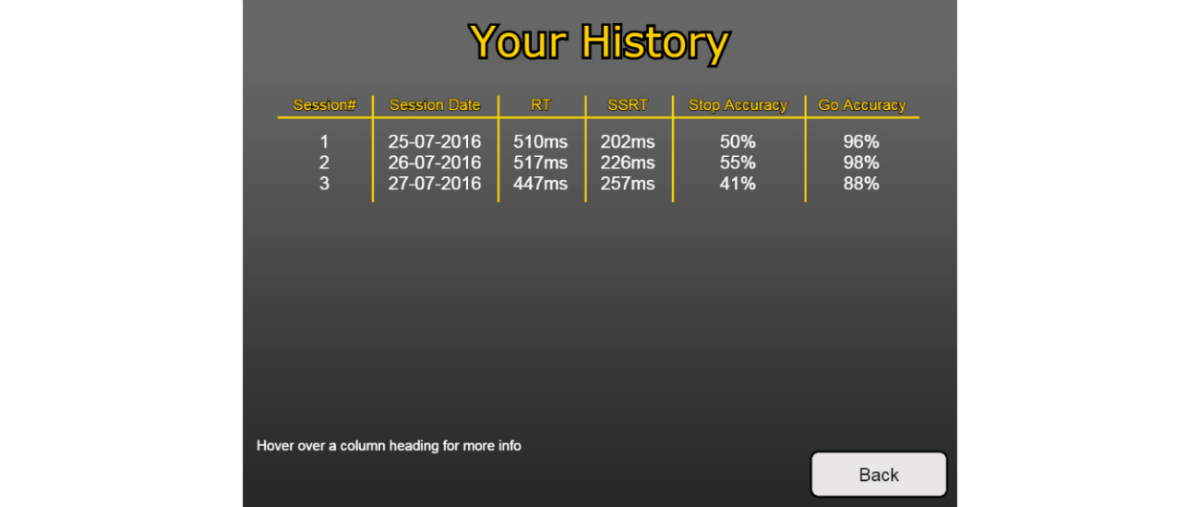
Screenshot of the nongame variant history screen.

In the nongame variant, the participant’s history was presented as a list of previous sessions, with median reaction times and estimated SSRTs (Figure 5). Hovering over a column displayed a brief explanation of the variable (eg, “The reaction time column shows the average time in milliseconds, which it took you to respond to the circles appearing in each session.”).

#### Stop Signal Task: Points Variant

**Figure 6:**
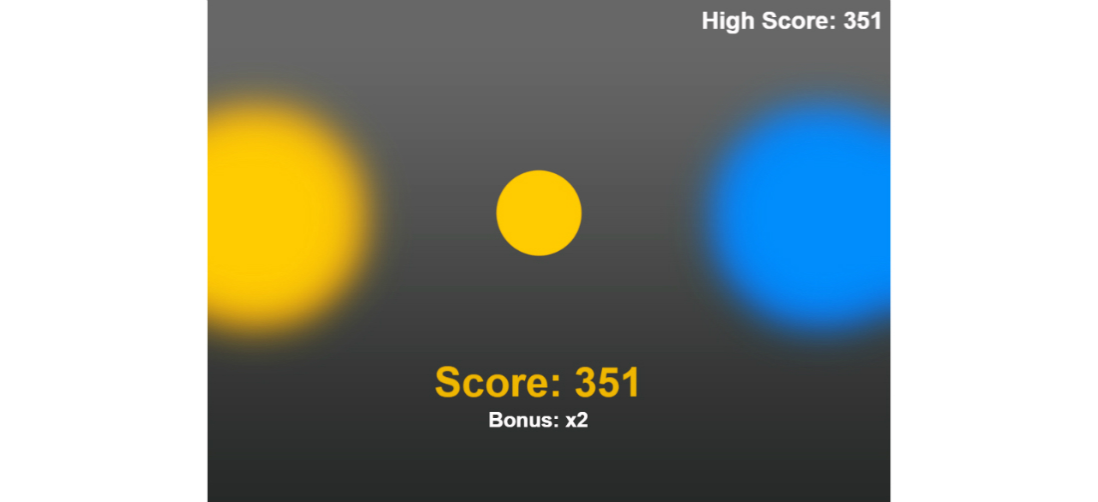
Screenshot of the points variant stop signal task.

The points variant was similar to the nongame variant but with the addition of a points mechanic and the task being framed as a game. Points are a common feature of gamified tasks [25] and are classed as “1st Step” gamification [57]. In our task, the participant’s points score was displayed at the bottom of the screen throughout (Figure 6) (Multimedia Appendix 3). The scoring system was very similar to that used in our previous study [43], which in turn was based on that used by Miranda et al [33]. The scoring system also incorporates the findings of Guitart-Masip et al [58] who found that subjects were much more successful in learning active (go) choices when rewarded for them and passive choices (stop) when punished. On each successful nonstop trial, the participant earned points equal to 0.2 × bonus × (800 - reaction time), and the number of points gained was displayed briefly in the intertrial interval. This bonus was a multiplier (×2, ×3, ×4…), which increased by 1 every 3 trials but decreased by 3 when the participant failed a stop trial. The bonus was not lost on stop trials to which the participant responded before the stop signal was displayed (to all appearances, the trial was not a stop trial). On a successful inhibition to a stop signal, the bonus was not lost, but no points were awarded (as there was no reaction time on which to base the score for that trial). Scores were maintained over blocks but not over sessions. The scoring system was outlined to the participants in the instructions for the task.

**Figure 7:**
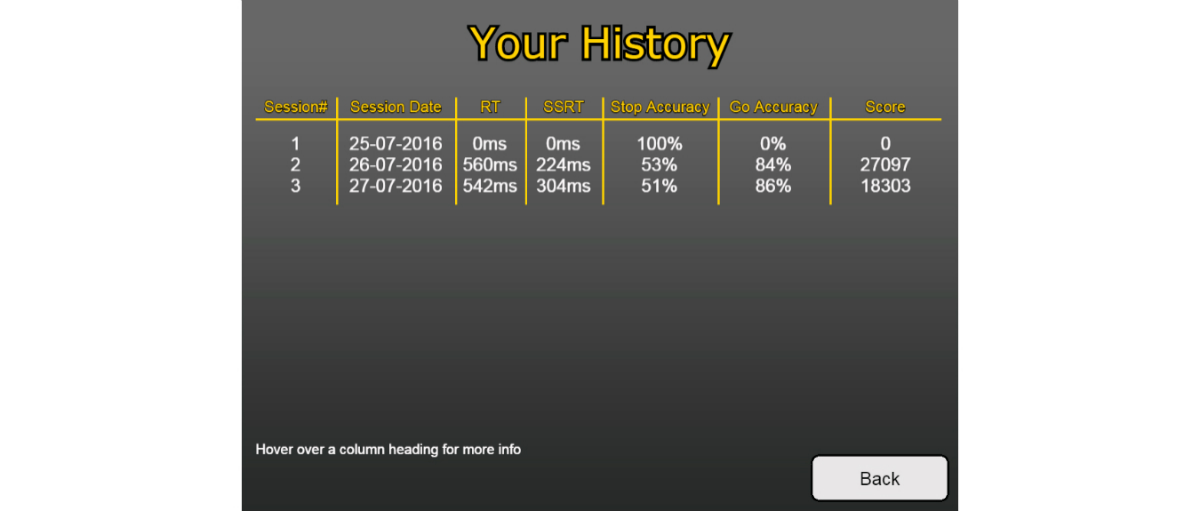
Screenshot of the points variant history screen.

The participant’s history was presented as a list of median reaction times, SSRTs, and scores from each testing session (Figure 7). Additionally, the participant’s highest score was saved as a high score and was displayed in the top right-hand corner throughout every testing session.

#### Stop Signal Task: Theme Variant

**Figure 8:**
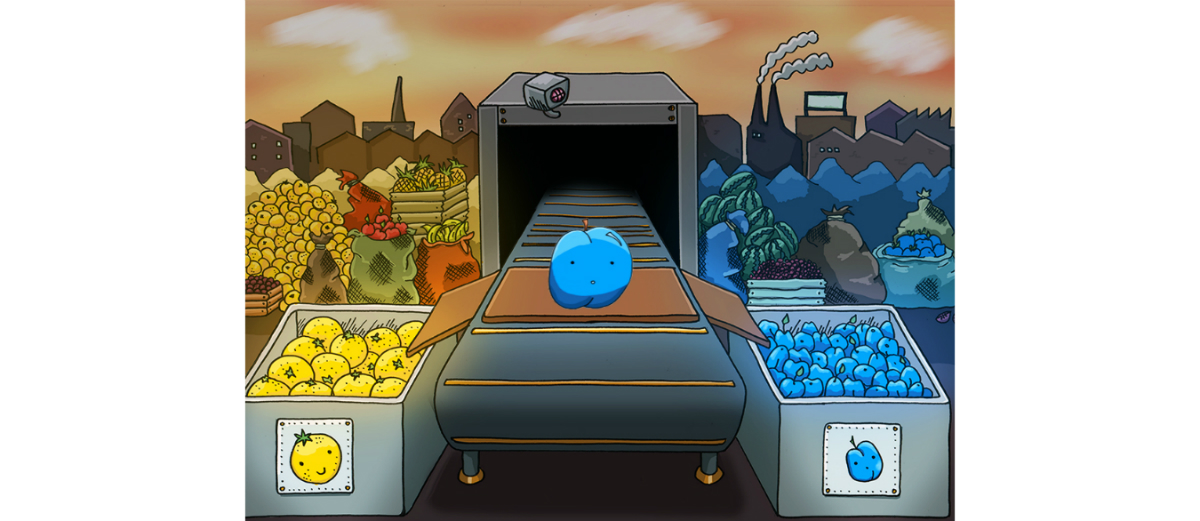
Screenshot of the theme variant stop signal task.

The theme variant was similar to the nongame variant but with the addition of a graphical theme and a sense of progression. The task was framed as a game and featured themed graphics and stimuli, with the yellow and blue stimuli being replaced by images of objects, though still predominantly blue or yellow (Figure 8, Multimedia Appendix 4). The task was presented on a series of different graphical backgrounds (Multimedia Appendix 5) but with some shared elements: a conveyor belt on which objects appeared and two bins to the left and right into which these objects were sorted. The stop signal was explained as an automatic *fault detector*, which scanned objects as they sat on the conveyor.

**Figure 9:**
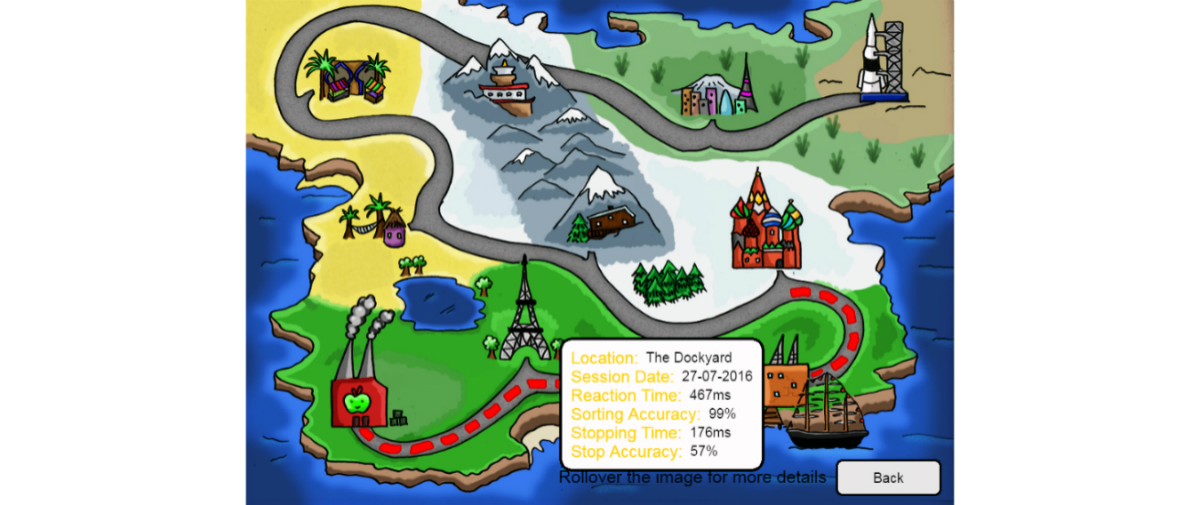
Screenshot of the theme variant history (map) screen.

The participant’s history was presented as a map (Figure 9), and previous sessions’ summary data were displayed when the user hovered over the corresponding icon. Each level on the map had a unique name and thematic instruction text, with the intention of creating an overarching goal, perceptual curiosity, and fostering a sense of participant progression [59-61].

#### Enjoyment and Engagement Questionnaire

The enjoyment and engagement questionnaire was designed to collect subjective ratings of the task and was delivered after every session for all three variants. Sessions 1, 4, 7, and 10 delivered the full 10-item questionnaire, whereas the remaining sessions delivered a shorter 5-item questionnaire. These items were answered using a continuous visual analog scale, presented as a horizontal line, 500 pixels long, with a label at either end and no subdivisions. Participants marked a point between these two labels using their mouse.

The following questions were based on those used in previous studies [31,33,43] and were presented in a random order: (1) How enjoyable did you find that?, (2) How frustrating did you find that?, (3) How difficult was it to concentrate for the duration of that?, (4) How well do you think you performed on that?, (5) How mentally stimulating did you find that to be?, (6) How boring did you find that?, (7) How much effort did you put in throughout that?, (8) How repetitive did you find that?, (9) How willing would you be to do that again tomorrow?, and (10) How willing would you be to recommend the study to a friend? Questions 3, 4, 6, 7, and 10 appeared only in the long version of the questionnaire.

### Dependent Variable Calculation

#### Attrition

Attrition was measured in two ways: First, we calculated the mean number of sessions completed per participant (sessions which were started but not finished were excluded from this calculation). Second, we calculated the percentage of participants that completed at least one session, two sessions, etc.

#### Subjective Measures of Engagement

Subjective engagement with the task was measured by calculating a mean score from the 10-item enjoyment and engagement questionnaire. Questions 2, 3, 6, and 8 were reverse-scored in this calculation. This measure was calculated for each participant’s first and fourth sessions, and we also created a *combined score* by taking the mean of the participant’s scores from sessions 1 and 4.

#### Objective Measures of Engagement

We piloted two measures that could potentially serve as objective proxies for engagement: we counted the number of times that participants hid the browser window or moved focus to another window while completing the SST, hypothesizing that unengaged participants would be more likely to briefly visit other websites while testing. We combined the counts of both these events into a single measure: loss-of-focus events. We then created an overall measure of loss-of-focus events for each participant by calculating the mean number of loss-of-focus events from their first four sessions.

We also investigated coefficients of variation, which quantify reaction time intra-individual variability with respect to mean reaction time, as there is some evidence that changes in motivation can be reflected in reaction time variation [62,63]. Coefficients of variation were calculated by dividing the standard deviation (SD) of nonstop trial reaction times by the mean nonstop trial reaction time. Similarly, we created an overall measure of reaction time variation for each participant by calculating the mean coefficient of variation from their first four sessions.

#### Stop Signal Reaction Times

We calculated SSRTs for each session separately, excluding sessions where the assumptions of the race model did not hold. The race model is a commonly used model of inhibitory control and aims to describe the relationship between stop and go processes [64]. The race model is used to derive the SSRT and so if the assumptions underlying the race model are broken, then the resultant SSRTs are not good representations of the data [50,64]. To that end, we excluded sessions where the median nonstop trial reaction time was longer than the median failed stop-trial reaction time, where SSDs were not positively correlated with their corresponding median failed stop reaction times, and where stop-trial accuracy was not negatively correlated with SSD.

For the sessions that did meet the assumptions of the race model, SSRTs were calculated by modeling an inhibition function, and using it to estimate the SSD at which the participant’s probability of inhibiting a stop signal was 50% [56]; we then used this SSD to calculate the SSRT for that session [50,51]. We also created a combined measure of SSRT for each participant by taking the mean SSRT of their first four sessions.

### Statistical Analysis

The data that form the basis of our results are available on request from the University of Bristol Research Data Repository [65].

#### Attrition

Differences in attrition curves were assessed visually using the Kaplan–Meier method to estimate survival functions, a log-rank test, and a one-way analysis of variance (ANOVA) of number of sessions completed.

#### Subjective Measures of Engagement

We assessed differences in subjective ratings both visually, using bar charts, and using a repeated measures ANOVA of the total score with session number as the time factor and task variant as the between-subjects factor. Where there was evidence of a difference between task variants, we used post hoc *t* tests to investigate further.

#### Objective Measures of Engagement

We assessed differences in coefficient of variation and website loss-of-focus events between task variants using one-way ANOVAs with data combined across the first four sessions. Where there was evidence of a difference between task variants, we used post hoc *t* tests to investigate further.

#### Stop Signal Reaction Times

We used boxplots and a one-way ANOVA with task variant as the between-subjects factor to investigate the effects of gamification on SSRT.

**Table 1:** Interpreting Bayes factors.

Hypothesis 0: the effect size is 0	Strength of evidence	Hypothesis 1: the absolute effect size is between 0 and X^a^
.33≤BF^b^≤1	No support either way	1≤BF≤3
.1≤BF≤.33	Positive	3≤BF≤10
.01≤BF≤.1	Strong	10≤BF≤100
BF<.01	Decisive	BF>100

^a^X: Cauchy prior width.

^b^BF: Bayes factor.

### Bayesian Analyses

The three task variants were designed with the aim of minimizing differences in primary task reaction time and nonstop trial accuracy. Therefore, given that frequentist statistics are not ideal for testing equivalences [66,67], we used Bayesian *t* test to assess the evidence for equality of means where frequentist methods failed to find a difference [68,69]. A Bayesian *t* test produces a Bayes Factor (BF), which compares the evidence for two hypotheses. If the evidence favors one hypothesis over the other, then the BF will reflect that, but if the evidence is equal for both hypotheses, then the BF will imply that the data are insensitive [69-71] (Table 1). We used the Bayesian *t* test procedure in JASP [72], with a Cauchy prior width of 0.707. Setting the Cauchy prior width to 0.707 means that in our analysis, one hypothesis is *the effect size is zero* and the other is *the effect size is between −* *0.707 and 0.707*. Although both hypotheses are centered on an effect size of 0, the former makes a stronger claim than the latter. As such, effect sizes that are not close to 0 are better represented by the latter hypothesis. A prior width of 0.707 was selected for our analysis because it represents the expectation of a medium-large effect, thus weighting the BF against small effects and reducing the likelihood of a false positive.

### Sample Size Determination

At the time of study design, to the best of our knowledge, no other studies had investigated the impact of gamification on attrition from a cognitive testing program, and therefore, we had no previous effect size on which to base a sample size determination. Instead, we hypothesized attrition curves (see Multimedia Appendix 1) for each variant and calculated the anticipated effect size (φ=0.231) resulting from a Kaplan–Meier method and log-rank test (ie, a chi-square test) on those attrition curves. To detect this difference with alpha=.05 and 95% power, a sample size of 290 was required. We set this to 291 to allow for equal group sizes.

## Results

### Characteristics of Participants

Participants were recruited in two waves: one starting in October 2016 and another starting in January 2017. In both waves, the intended sample size was met within 3 days of the study being posted on Prolific Academic. A total of 482 participants signed up to take part in the study, with 419 (86.9%) of those completing at least one session. A total of 265 (54.9%) participants completed four sessions over 4 consecutive days as was required by the study criteria (henceforth called *conforming participants*). We excluded 5 participants from the analysis because their reaction times or stimulus categorization accuracy scores were more than 4 interquartile ranges away from the group median. We excluded data from sessions that were started but not completed, and we removed trials from the analysis where participants responded in less than 150 ms.

The analysis below presents data from 260 participants, that is, less than our intended sample size of 291. This was because 32 participants failed to complete the required four sessions in 4 days but instead managed to complete four sessions within 5 days. During the study, we intended on including these *loosely conforming* participants in the analysis, and so stopped recruitment once our intended sample size was achieved. However, for simplicity and adherence to the protocol, we have now decided to present only strictly conforming participant’s data below. Analysis of the nonconforming and loosely conforming participants’ attrition is presented in Multimedia Appendix 1.

Excluding outliers, 260 conforming participants took part: 91 in the nongame variant, 86 in the points variant, and 83 in the theme. The number of hours spent playing video games was comparable between the groups, and participants typically had a high level of education (Table 2). The most common browser used to complete the experiment was Google Chrome (n=184, 70.8%), with others including Firefox (n=50, 19.2%), Netscape (n=13, 5%), Safari (n=11, 4.2%), Opera (n=1, 0.5%), and Internet Explorer (n=1, 0.5%).

### Attrition

**Figure 10:**
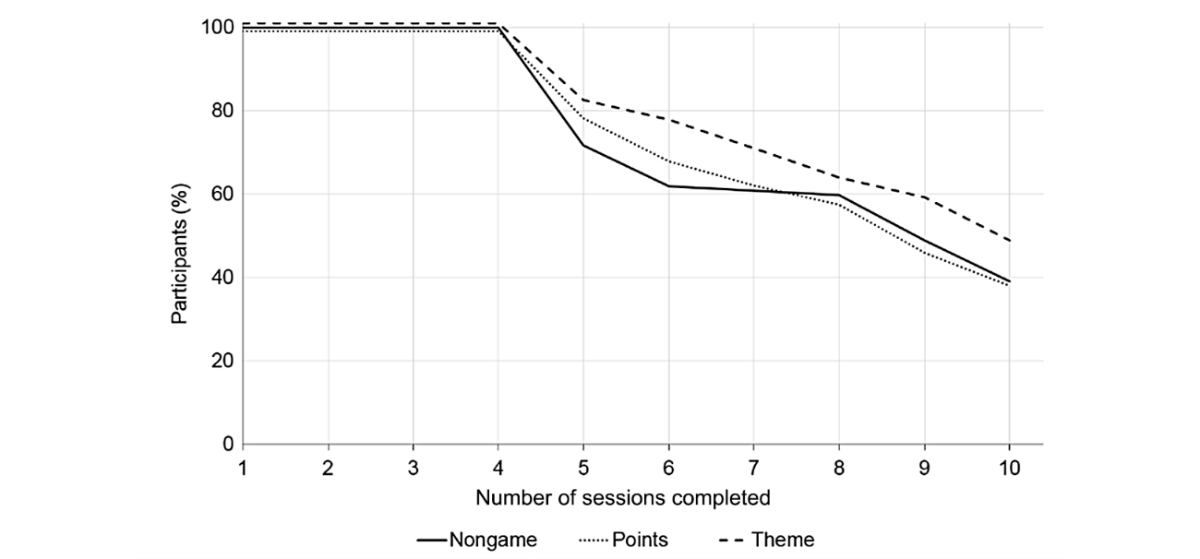
Percentage of conforming participants plotted against the number of sessions they completed, shown separately by task variant.

Figure 10 shows the attrition of conforming participants, whereas Table 3 shows the mean number of sessions completed per participant in each variant. A log-rank test showed no evidence of a difference between the distributions (χ^2^_2_=3.0, *P*=.22), and a one-way ANOVA of the number of sessions completed also found no clear evidence of a difference between task variants (*F*_2,262_=1.534, *P*=.21, partial η^2^=0.012). Given the similarity between nongame and points in mean number of sessions completed, we used a Bayesian *t* test to assess their equality and found substantial evidence that they were equal (BF=0.16), but there was no evidence of equality between the theme and the points variant (BF=0.49) or the nongame variant (BF=0.43).

**Table 2:** Conforming participants’ demographic information, shown separately by task variant.

Demographic	Nongame	Points	Theme
Age, mean (SD)	36 (12)	35 (12)	34 (11)
Male, n (%)	43 (47)	49 (57)	42 (51)
Mean video game hours per week (SD)	6 (12)	8 (16)	8 (14)
Median level of education	Bachelor’s degree	Bachelor’s degree	Bachelor’s degree
Mode ethnicity, n (%)	White, 80 (88)	White, 74 (86)	White, 75 (90)

**Table 3:** Mean number of sessions completed per participant, shown separately by task variant. Conforming participants are those who completed their first four sessions within 4 days as required. All participants includes all who signed up, regardless of their number of sessions completed.

Variant	All participants (95% CI)	Conforming participants (95% CI)
Nongame	4.9 (4.4-5.5)	7.4 (6.8-8.0)
Points	5.1 (4.5-5.6)	7.5 (7.0-8.0)
Theme	5.3 (4.7-5.9)	8.0 (7.5-8.6)

### Subjective Measures of Engagement

**Figure 11:**
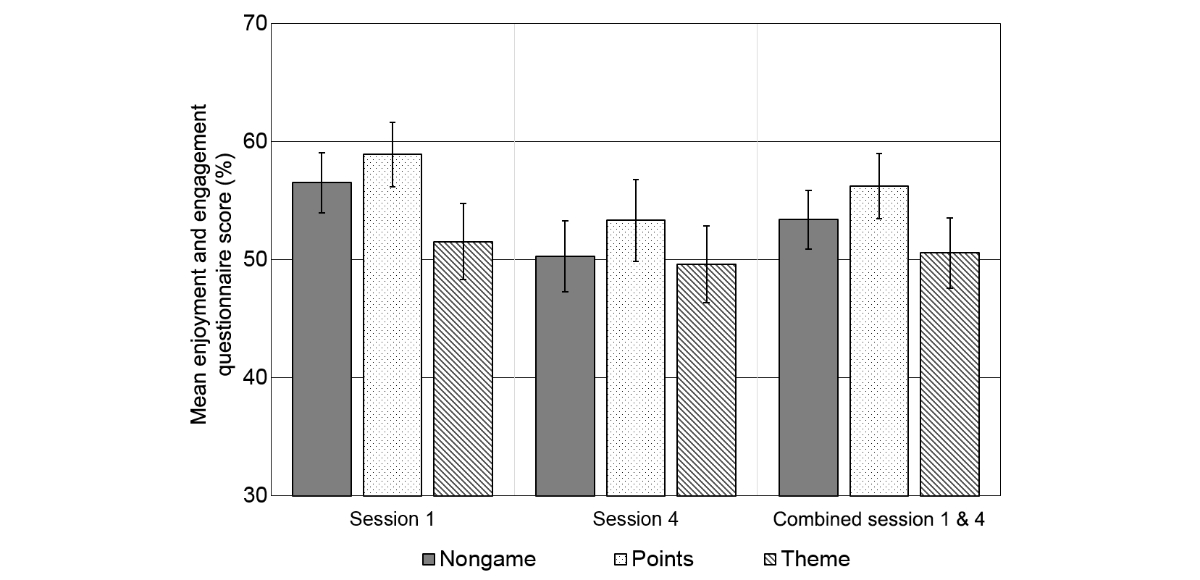
Overall scores from the subjective enjoyment and engagement questionnaire. Mean responses of visual analog scale scores from questionnaires delivered on sessions 1 and 4, and the average scores from sessions 1 and 4, shown separately by task variants and time point. Error bars represent 95% CIs.

We used a repeated measures ANOVA of mean score from the enjoyment and engagement questionnaire with session number (1 and 4) as the within-subjects factor and task variant as the between. We used only the two full-length questionnaires completed on the first and the fourth sessions and completed by all participants (for short-form questionnaire results, see Multimedia Appendix 1). We saw evidence for small effects of both task variant (*F*_2,261_=3.805, *P*=.02, partial η^2^=0.028) and time (*F*_1,261_=35.693, *P*<.001, partial η^2^=0.120), and weak evidence of an interaction (*F*_2,261_=3.014, *P*=.05, partial η^2^=0.023). We noted that ratings of all task variants decrease between the first (M=56, 95% CI 54-57) and fourth sessions (M=51, 95% CI 49-53), but it appears that the nongame and points variants were the main drivers of the interaction effect: dropping by 6% (95% CI 4-8) between sessions 1 and 4, whereas ratings of the theme task decreased only by 2% (95% CI −1 to 5). Post hoc *t* tests on the combined scores showed no evidence for differences between nongame and points, or for nongame and theme (*ps*>.15) but did show points and theme to be different (mean difference=5.7%, 95% CI 1.6-9.7, *t*_171_=2.749, *P*=.01, *d*=0.42). Figure 11 shows the mean scores from each task variant at two time points and a combined score taking the averages of both sessions. A breakdown of ratings by individual questions is presented in Multimedia Appendix 1.

As an unplanned exploratory analysis, we were interested in whether a participant’s rating on one day predicted their return to the study on the following day. We ran a logistic regression with *returned following day* as the binary dependent variable and the previous day’s score on the subjective questionnaire as the predictor variable. However, we saw no evidence that subjective questionnaire scores predicted return the following day (beta=.008; standard error=0.005; Wald_1_=2.166; *P*=.14; odds ratio=1.001, 95% CI 0.997-1.019).

### Objective Measures of Engagement

We analyzed reaction time coefficient of variation and website loss-of-focus events from the four compulsory sessions combined (Table 4). A one-way ANOVA of coefficient of variation showed strong evidence for a medium effect of task variant (*F*_2,260_=3.131, *P*=.045, partial η^2^=0.024) on participants’ reaction time variability, with lower coefficients indicating that there was less variability. Post hoc *t* tests showed strong evidence of a difference between the points and theme variants (mean difference=1.5%, 95% CI 0.2-2.7; *t*_170_=2.349; *P*=.02; *d*=0.36), but no clear evidence for other differences were found (*ps*>.06).

Loss-of-focus events were rare in all task variants, with each participant switching away from the task less than once per session on average. Regardless, we assessed differences in loss-of-focus events between the three task variants using a one-way ANOVA but found no evidence of any difference (*F*_2,260_=1.137; *P*=.32; partial η^2^=0.008).

### Stop Signal Reaction Times

**Figure 12:**
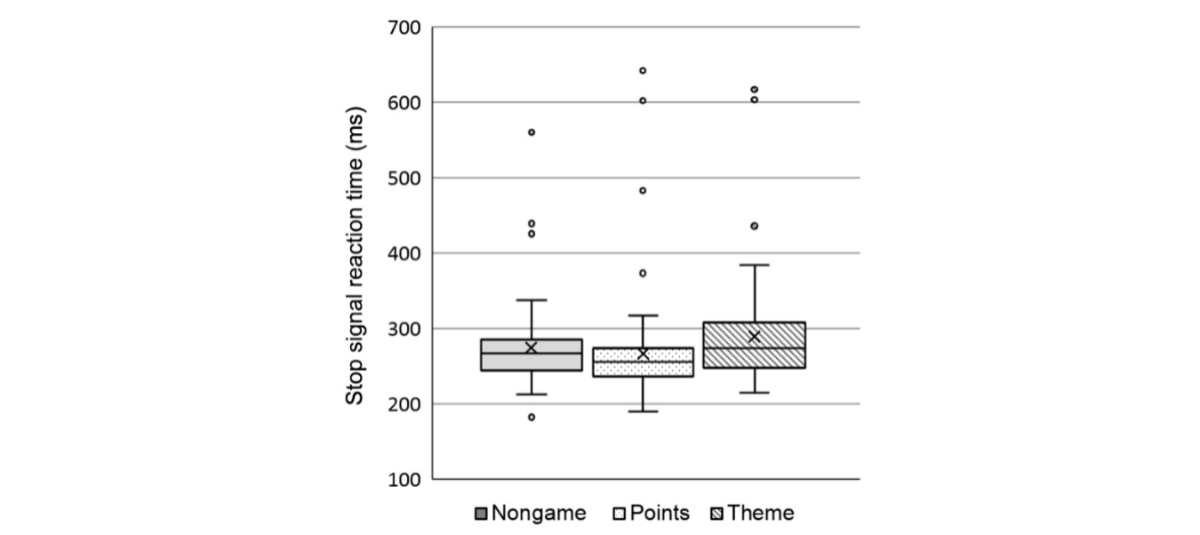
Boxplots of mean stop signal reaction time. Data combined per participant over the first four sessions and shown separately by task variants.

We checked the data from each session against the assumptions of the race model. Of the 1050 sessions assessed, we excluded 161 sessions: 75 from the nongame variant, 37 from points, and 49 from theme. A total of 3 participants failed to meet the assumptions of the race model in all four compulsory sessions, resulting in their exclusion from this analysis. We then analyzed each participant’s mean SSRT, with boxplots shown in Figure 12.

A one-way ANOVA showed weak evidence for a small effect of task variant on SSRT (*F*_2,255_=2.954; *P*=.05; partial η^2^=0.022) with post hoc *t* tests showing a difference between the theme variant (M=289; SD=67) and points variant (M=266, SD=66; mean difference=23, 95% CI 5-42; *t*_169_=2.386; *P*=.05; *d*=0.35). There was no evidence for other differences (*ps*>.24). Bayesian *t* tests showed no evidence of equality between the SSRTs of the nongame and theme variants (BF=0.59) but found substantial evidence for equality between the nongame (M=274, SD=55) and the points variants (BF=0.22).

For brevity, not all the analyses planned in the study protocol have been presented. For more detailed methods and analyses, please see Multimedia Appendix 1.

**Table 4:** Mean objective measures of participant engagement from the first four sessions, shown separately by task variant.

Variant	Coefficient of variation (%) (95% CI)	Loss-of-focus events (95% CI)
Nongame	18.7% (17.9-19.6)	0.85 (0.50-1.19)
Points	19.0% (18.1-19.8)	0.82 (0.43-1.20)
Theme	17.5% (16.7-18.4)	1.21 (0.75-1.67)
Overall	18.4% (17.9-18.9)	0.95 (0.72-1.18)

## Discussion

### Principal Findings

Contrary to our hypotheses, we saw no clear evidence of an effect of task variant on participant attrition. This was further strengthened when we included data from loosely conforming participants (see Multimedia Appendix 1), which showed strong evidence that the mean number of sessions completed was equal in all task variants. To the best of our knowledge, this is the first empirical study examining the effects of gamification on participant attrition within a cognitive testing context, and our results raise doubts about the efficacy of gamelike tasks for reducing participant dropout.

Despite there being no difference in usage between the variants, we did find an effect of task variant on the subjective ratings of the tasks, with the points variant having the highest *combined sessions* mean, followed by the nongame variant and the theme. One possible explanation for these findings relates to self-determination theory, a popular theory of motivation that centers around the concept of psychological needs and need satisfaction. Self-determination theory posits that human beings have three needs, which are competence, autonomy, and relatedness and that we find activities to be intrinsically motivating if they help us to fulfill these needs [73]. In the case of our gamelike variants, the points variant would seem to address competency needs by providing constant feedback on their performance, which reinforces the player’s success [74], but we do not consider the theme variant or the nongame variant to adequately meet any of the three needs. As the points variant was the only variant to address any of these needs, this may explain why it was rated as the most enjoyable in both this study and our previous study [43].

The theme variant was rated as the worst of the three tasks, which was surprising as it maintained the highest percentage of participants until day 10. One potential explanation is that the task was framed as a game and looked like a game but offered no actual gameplay. Moreover, the map screen and changing graphical backgrounds may have hinted at player autonomy and exploration as is typical in other games, but ultimately, the player experience was railroaded. These two factors may have undermined autonomy and violated participant expectations, resulting in a dissatisfying experience [75,76]. Despite this, it is possible that the clear end goal on the map and novelty of changing backgrounds could explain the maintenance of participants in the theme variant while still not being a very satisfying or enjoyable experience.

One additional factor to consider, in the light of self-determination theory, is that paying participants in attrition studies such as this may be counterproductive to measuring true engagement. There is evidence that providing extrinsic rewards for otherwise motivating tasks may undermine participant autonomy, therefore affecting the task’s ability to meet our psychological needs [77,78]. In this study, it is not possible to determine whether intrinsic motivation to take part was affected by the incentive of 50p per additional session. This is further complicated by the potentially unrepresentative nature of a Prolific Academic sample: all of whom have voluntarily signed up to take part in science experiments over the Internet but can choose studies based on the amount of monetary compensation awarded in exchange for their data. Given these issues, one potentially informative avenue for future research in this area would be to explore the effects of these same gamification mechanisms on attrition but without providing financial incentives.

Money can be a powerful motivator; for example, Khadjesari et al [79] found that offering a £10 Amazon voucher to each participant in a longitudinal study resulted in a 9% increased response rate. In this study, it may simply be that money was the most important factor for taking part and that the similar attrition rates were driven by the identical incentives.

We also found no evidence that participant ratings of engagement and enjoyment could predict the number of optional sessions they would complete. This, combined with the disconnect between the theme variant ratings and theme variant usage, serves to highlight the split in different types of engagement that has begun to be conceptualized in the literature [24]. In short, the word engagement has been used in the past to refer to both *engagement as subjective experience* and *engagement as usage*, and this study is further evidence that the two concepts are not as closely related as one might assume. Evidence from the video game literature has found that game enjoyment does not relate strongly to game usage, and that game usage can be driven by many other factors, including boredom, loneliness, and need for escapism [80,81]. This highlights the need for future studies of engagement, which collect both subjective and objective measures.

Our two pilot objective measures of engagement, reaction time variation (coefficients of variation) and loss-of-focus events, were difficult to interpret. We saw no evidence that losses of focus differed between the task variants, and this is likely because such events were rare (less than one loss-of-focus event per session on average). This is a positive finding, as it shows that participants are willing to fully concentrate on Web-based cognitive tasks. With respect to coefficients of variation, the pattern of results is directly in contrast with our subjective measures of engagement: the points variant had the most variable response times but the highest subjective rating, whereas the theme variant had the lowest variability and the lowest rating. This is either evidence contrary to the premise that reaction time variability is related to motivation [62,63] or signals that our subjective ratings are not good measures of motivation. Regardless, further research is necessary to understand whether these objective measures provided are related to the broader concept of engagement.

When assessing cognitive data, we found evidence that SSRTs were equivalent between the points variant and the nongame variant. Although the points variant introduced additional elements to the task, which may have increased cognitive load, it is possible that the highly salient feedback and motivational effect of points served to increase participant performance as has been found in a number of previous studies [41,82-84].

### Limitations and Conclusions

First, we consider the fact that we did not achieve our intended sample size, an important limitation of this study. However, we maintain that the results of our supplementary analyses including the loosely conforming participants are quite conclusive and strengthen our finding that there was no effect of gamification on attrition. However, we accept that a balanced group analysis would be preferable. Second, we acknowledge that our sample, recruited from Prolific Academic, with high levels of education, may not be representative of the wider population. Third, we acknowledge that the design of study used is not suitable to validate our gamelike variants as measures of response inhibition, as that would require a within-subjects design to test predictive validity [57,85]. Fourth, the gamelike features we implemented were very lightweight and certainly would not constitute a full game. Indeed, neither of our games were likely enjoyable enough that a participant would consider doing them for their own sake. Though this was necessary to try to reduce the impact of gamification on the cognitive data, it likely reduced any effect of gamification that we might have seen. Fifth, the time course of our study, which took place over days, may not be informative about attrition in studies that take place over longer periods of weeks or months. Sixth, as mentioned previously, there are issues relating to motivation and incentives, as in reality, participants completing cognitive assessments will be presented with requests to complete a study over a fixed period for a fixed fee and not with the option to continue for additional recompense. Finally, both incentives and reminders have been well established as effective methods of increasing engagement, and we used both in this study [86]. Although all three task variants had the same incentives and the same program of reminders (which stopped on day 4), it is possible that these baseline engagement strategies acted as confounders, potentially muddying the effect of gamification on attrition.

In conclusion, the theme variant had negative effects on the cognitive data and showed no clear evidence of reducing attrition. It was also rated as the least enjoyable and was the task switched away from most often. This suggests that themed gamelike tasks, at least those that use graphics alone, are nonoptimal for use in cognitive assessment studies. In contrast, and replicating our previous finding [43], subjective ratings showed the points variant to be well received. We found SSRTs from the points and nongame variants to be equal, showing that points can be an effective way of increasing participant enjoyment of a cognitive task while still collecting valid data.

Despite differences in subjective ratings between the task variants, we saw no effect of gamification on participant attrition over the 6-day optional testing period. Gamification has been promoted as a potential solution to engagement problems in both psychology and digital health care for several years, but we found no effect of gamification on *engagement as usage* in this case. The term gamification may have existed for a decade, but the formalization of gamification’s implementation and effectiveness is only just beginning, and there is clearly further work to be conducted to understand how we can translate differences in subjective ratings to differences in usage.

## Appendix

Multimedia Appendix 1 A document containing additional details on the methods used in the study and supplementary analyses not core to the message of the paper.

Multimedia Appendix 2 Animated GIF showing several trials of the Non-Game variant of the Stop Signal Task.

Multimedia Appendix 3 Animated GIF showing several trials of the Points variant of the Stop Signal Task.

Multimedia Appendix 4 Animated GIF showing several trials of the Theme variant of the Stop Signal Task.

Multimedia Appendix 5 PDF document showing all possible task backgrounds from the theme variant.

## Abbreviations

ANOVA: analysis of variance
BF: Bayes Factor
SD: standard deviation
SSD: stop signal delay
SSRT: stop signal reaction time
SST: Stop Signal Task
