# Supplementary material for: Attrition from Web-Based Cognitive Testing: A Repeated Measures Comparison of Gamification Techniques
Source: J Med Internet Res. 2017 Nov 22;19(11):e395. doi: 10.2196/jmir.8473 (PMC5719230; doi:10.2196/jmir.8473)
Supplement: Multimedia Appendix 1 [file jmir_v19i11e395_app1.pdf]

Supplementary Methods:

Attrition Hypotheses

We expected the non-game variant to have the highest attrition rate, losing participants quickly after the fourth compulsory session was complete. We anticipated this because the Non-game variant contained no gamelike features; therefore, the only motivating factor was the reward of 50p per session. A pilot study showed that the Non-Game variant with a reward of 50p per session had only 63% of its participants complete the first optional session and only 38% complete all six optional sessions.

We expected the Points variant initially to maintain high numbers, before falling rapidly around day 6-7. We assumed this would happen as participant performance plateaued and high scores could no longer be easily broken, and as the "pointlessness" of the points became apparent. This hypothesis is based on the premise that Points are extrinsic motivators and thus suffer from diminishing returns when used in isolation (Mekler, Brühlmann, Opwis, & Tuch, 2013; Mekler, Brühlmann, Tuch, & Opwis, 2015).

We expected the Theme variant to lose participants steadily at first before stabilizing to a low attrition rate, eventually retaining a higher number of participants than either the Non-Game or Points variants. We expected the map would encourage participants to see the study through to its conclusion by providing a sense of progression (Malone, 1981). Furthermore, we expected the variety in task backgrounds to add novelty to each test session, thereby motivating participants to return to the study the next day (Malone, 1980).

Based on these hypotheses, we generated hypothetical attrition curves (see Supplementary Figure 1). We then used these curves to estimate an effect size and perform a sample size calculation.

Supplementary Figure 1: Hypothesised percentage of participants completing a session on each day over the 10-day period.

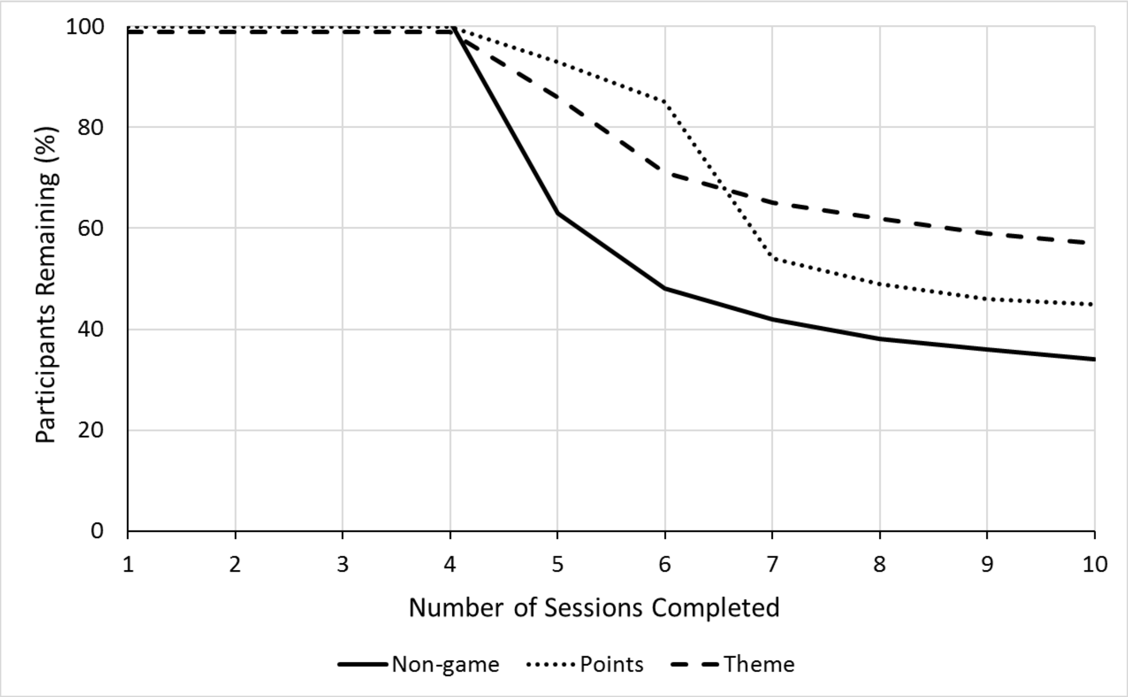

Procedure

The study elements participants completed each session depended on the day of the study they were currently on. From the main menu, clicking the start button would display a series of instructions screens followed by a ~10-minute delivery of the SST and that day's questionnaires, as shown in Supplementary Table 1.

Supplementary Table 1: Study elements delivered each session. Optional sessions are shown in light grey

	Session Number									
	**1**	**2**	**3**	**4**	**5**	**6**	**7**	**8**	**9**	**10**
**Instructions**	X	X	X	X	X	X	X	X	X	X
**Stop Signal Task**	X	X	X	X	X	X	X	X	X	X
**Demographic Questionnaire**	X									
**Full Engagement Questionnaire**	X			X			X			X
**Short Engagement Questionnaire**		X	X		X	X		X	X	
**Perseverance Questionnaire**		X								
**Free Text Questionnaire**			X							X
**History Screen**	X	X	X	X	X	X	X	X	X	X

Stop Signal Task: Staircase and Block Details

Further to the information provided in the main manuscript: Stop Signal Delay (SSD) was varied according to a four-staircase convergence algorithm, designed to sample evenly across the SSD/Inhibition-Probability space. Staircases 2 and 3 converged to a 50% failed inhibition rate, while staircases 1 and 4 sampled the limits of a participant's inhibition, see Supplementary Table 2. On a step-up or step-down a staircase was adjusted by +/-50 ms respectively, and the step size changed to +/-25ms after two reversals of direction. The shortest possible SSD was 25ms and the longest possible was 750ms.

Supplementary Table 2: Stop signal delay staircase initial values. Due to staircases 1 and 4 tracking the lower and upper limits of inhibition respectively, they require a different number of failed/successful inhibitions to step up or down.

**Staircase number**	**Initial SSD**	**Failure rate goal**	**Number of failed trials in a row needed to step down**	**Number of successful trials in a row needed to step up**
1	150ms	~30%	1	2
2	250ms	~50%	1	1
3	350ms	~50%	1	1
4	400ms	~70%	2	1

The task consisted of 5 blocks of 48 trials each. Each block contained 3 sub-blocks of 16 trials each, of which 12 were go trials and 4 were stop trials. The first sub-block of each session consisted entirely of Go trials, so in total each session contained 240 trials, of which 56 were stop trials. After 48 trials the block ended and the subject had to wait for 10 seconds before they automatically continued to the next block. In order to maintain response speed and to discourage strategy, the subject was prompted to go faster during this break. A dynamic speed-prompt was also displayed if the subject's responses in one sub-block were on average 50 ms slower than those in the previous sub-block. Once five blocks had been completed, the task ended. This typically took ~10 minutes.

Stop Signal Reaction Time Calculation
Estimated SSRTs were calculated automatically at the end of each session using the integration method as detailed in (Band, van der Molen, & Logan, 2003; Logan, 1994), and were presented to participants on the history screen.

Free Text Questionnaire
After the third session, participants were presented with a short questionnaire to which they could respond to using free text of up to 500 characters. The following questions were presented in a random order: (1) Have you noticed any bugs or errors in the experiment so far? (2) Are you enjoying the experiment so far? Is there anything you would change? (3) What has motivated you to take part in the experiment so far?

Perseverance Questionnaire
After the second session participants completed a visual-analogue-scale based perseverance subscale of the Urgency, Premeditation, Perseverance and Sensation Seeking (UPPS) Impulsive Behaviour Scale (Whiteside & Lynam, 2001), presented in the same format at the Enjoyment and Engagement questionnaire. The main aim of this questionnaire was to test whether individual differences in perseverance might confound attrition rates on the task variants. A total perseverance score was calculated as the mean of all items, with items 2 and 10 reverse-scored. The following questions were presented in a random order: (1) I generally like to see things through to the end, (2) I tend to give up easily, (3) Unfinished tasks really bother me. (4) Once I get going on something I hate to stop. (5) I concentrate easily. (6) I finish what I start. (7) I'm pretty good about pacing myself so as to get things done on time. (8) I am a productive person who always gets the job done. (9) Once I start a project, I almost always finish it, and (10) There are so many little jobs that need to be done that I sometimes just ignore them all.

Supplementary Analyses:
Planned Analyses
The following analyses were planned as described in our preregistered study protocol: https://osf.io/ysaqe/

Cognitive Data
Go RTs and FailedStop RTs were summarised at a participant level using medians. We also calculated the gradient of the inhibition function at the point of P(Respond|Signal) = 0.5 using numerical differentiation.

To assess whether the introduction of game mechanics would affect the cognitive data collected by each task variant we used mean Go RT, FailedStop RT, Go Accuracy and Stop Accuracy data from the four compulsory sessions and performed a series of univariate ANOVAs with task variant (Non-Game, Points, Theme) as a factor, see Supplementary Table 3. We found effects of task variant on all measures except for Go Accuracy, and this is likely because Go Accuracy scores were high and participants were operating at ceiling. The effects of task variant were quite small, yet still indicate an impact of game mechanics on the comparability of the data collected by the task.

Supplementary Table 3: Effects of task variant on Go Reaction Time, FailedStop Reaction Time, Go Accuracy and Stop Accuracy. Four univariate ANOVAs on cognitive measures from the first four sessions, with task variant (Non-Game, Points, Theme) as a between-subjects factor.

Dependant Variable	F[2, 260]	p	partial η^2
Go RT	4.421	0.014	0.032
FailedStop RT	5.403	0.005	0.040
Go Accuracy	1.053	0.350	0.008
Stop Accuracy	4.450	0.013	0.033

Supplementary Figure 2 shows boxplots of these variables for each task variant, made of up participants' median responses over the four compulsory sessions. Cognitive measures appear broadly comparable

between task variants, but the effects detected by the ANOVAs are apparent on closer inspection. We used t-tests to explore differences of interest, and Bayesian t-tests to assess similar distributions for equality.

Supplementary Figure 2: Box and whisker plots of mean Go Reaction Time, FailedStop Reaction Time, Go Accuracy and Stop Accuracy. Data combined per participant over the first four sessions and shown separately by task variant.

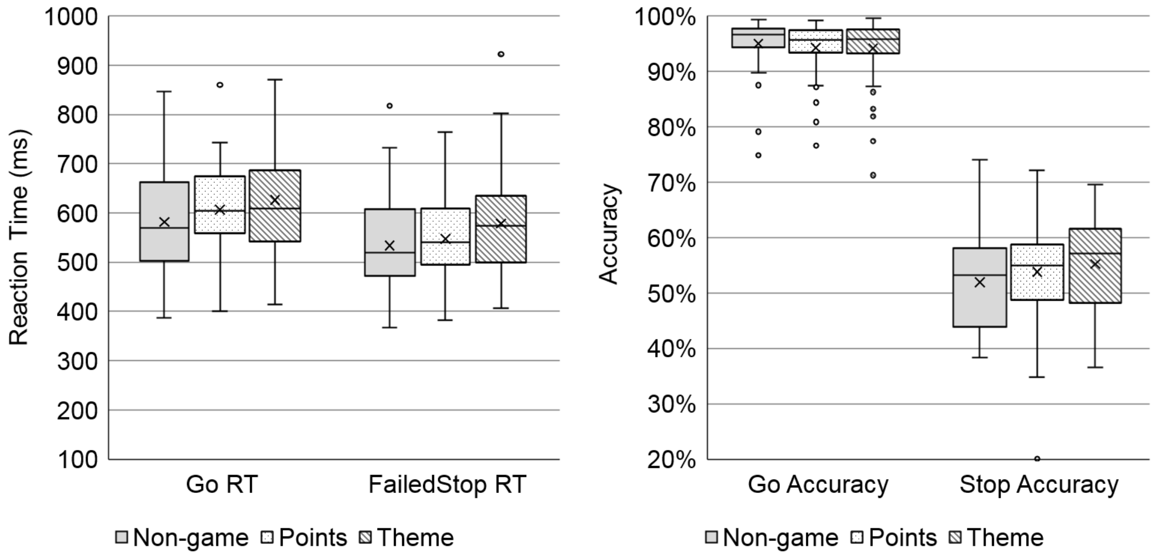

We found evidence of a difference in Go RT between the Non-Game (M = 583, SD = 100) and Theme (M = 622, SD = 92) variants (mean difference = 38, 95% CI 10 to 67, t(174) = 2.651, *p* = .01, *d* = 0.41), but no evidence of other differences (*p*s > .12). A Bayesian t-test for equality in Go RT distributions between the Non-Game and Point variants was inconclusive (BF = 0.89).

We also found evidence of a difference in FailedStop RT between the Non-Game (M = 530, SD = 88) and Theme (M = 570, SD = 87) variants (mean difference = 40, 95% CI 16 to 65, t(174) = 3.068, *p* = .01, *d* = 0.46), but little evidence of other differences (*p*s > .10). Again, a Bayesian t-test could not provide evidence of equality between the Non-Game and Points variants (BF = 0.42).

Given the lack of effect of task variant on Go Accuracy we used Bayesian t-tests to assess the variants for equality. These tests were inconclusive for all comparisons (BF = 0.31 and 0.38) except Points (M = 94%, SD = 5%) compared to Theme (M = 94%, SD = 6%), where we found substantial evidence of equality (BF = 0.17)

With respect to Stop Accuracy we saw differences between the Non-Game (M = 52%, SD = 9%) and Theme (M = 54%, SD = 7%) variants (mean difference 25%, 95% CI 21 to 48, t(169.9) = 2.153, *p* = .03, *d* = 0.32) and the Non-Game and Points (M = 55%, SD = 8%) variants (mean difference = 33%, 95% CI 9 to 57, t(175.2) = 2.722, *p* = .01, *d* = 0.40). There was no evidence of a difference between Points and Theme (*p* = .50), but a Bayesian t-test could not provide evidence for equality either (BF = 0.50).

Supplementary Table 4: Mean Stop Signal Reaction Times from the first four sessions, shown separately by task variant

	SSRT (95% CI)	Gradient of Inhibition Function at P(Respond|Signal)=0.5 (95% CI)
Non-Game	274ms (262 to 285)	-5.36% (-5.10 to -5.64)
Points	262ms (250 to 275)	-5.10% (-4.87 to -5.32)
Theme	286ms (273 to 299)	-5.22% (-5.05 to -5.38)

We also calculated the slopes of the modelled inhibition curves using numerical differentiation, and assessed the gradient for differences between task variants. A one-way ANOVA did not show evidence of an effect of task variant on inhibition slope (F [2,255] = 1.437, p = .24, *partial* η^2 = 0.011), and Bayesian t-tests showed moderate evidence that the Non-Game and Theme variants' slopes were equivalent (BF = 0.24), and that Points and Theme variants' slopes were also equivalent (BF = 0.22) However, there was insufficient evidence to suggest that the Non-Game variant and the Points variant had equivalent slopes (BF = 0.62), see Supplementary Table 4 and Supplementary Figure 3.

Supplementary Figure 3: Box and whisker plots of mean Stop Signal Reaction Time and mean Inhibition Function gradient. Data combined per participant over the first four sessions and shown separately by task variant

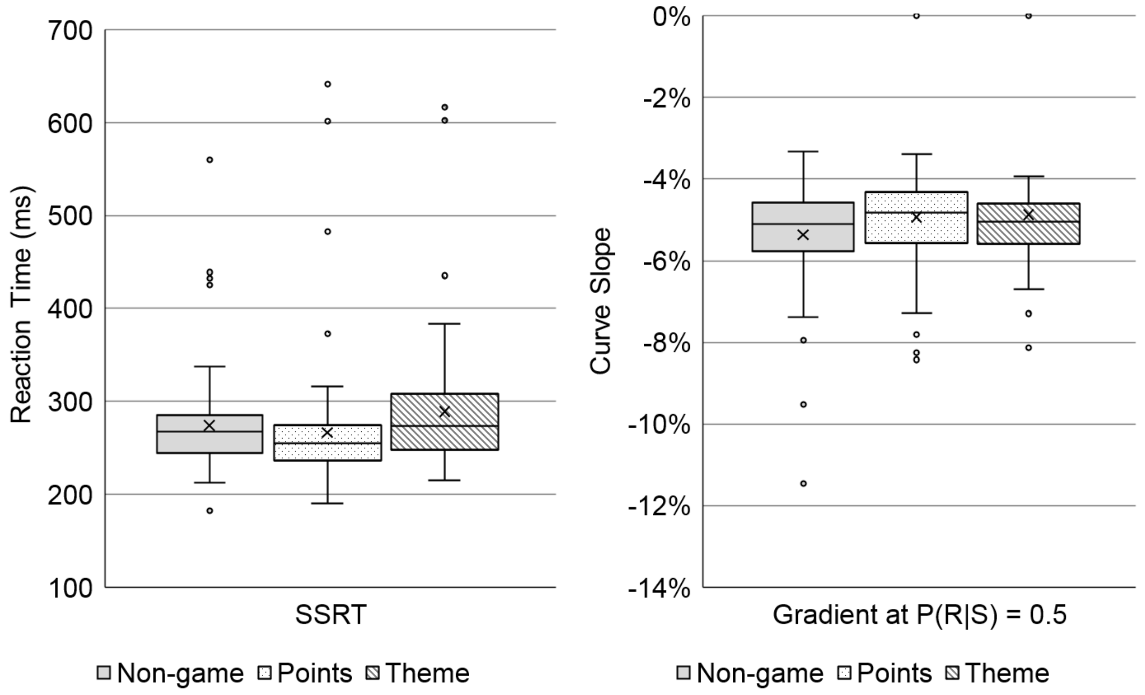

Reliability of Cognitive Measures over Time

We found the test-retest reliability of SSRTs from the first four sessions to be very good, with an overall Cronbach's alpha of 0.85. When assessed by task variant, the Points (α = 0.86), and Theme (α = 0.86) variants showed the most consistent results with Non-Game (α = 0.75) showing lesser, yet still good, reliability. We used *cocron* (Diedenhofen & Musch, 2016) to investigate differences between these alphas but saw no evidence for an effect of task variant (X^2 (2, N = 258) = 5.140, p = .08).

We also wanted to investigate whether time or practice effects impacted the cognitive data collected by the task variants, and so ran a series of repeated-measures ANOVAs with Go RT, FailedStop RT and SSRT as the dependant variables and session number (1-4) as the time factor in each, see Supplementary Table 5.

Where there was evidence of Sphericity we used Greenhouse-Geisser corrected *p* values. We saw small effects of session number on all cognitive measures, but no clear evidence of interactions between task variant and session number on any of the measures (*p*s > .07). Supplementary Table 6 shows the mean RTs from each session, combined across task variant.

Supplementary Table 5: Effect of session number on Go Reaction Time, FailedStop Reaction Time and Stop Signal Reaction Time. Three repeated-measures ANOVAs with session number (1-4) as the time-factor and task variant (Non-game, Points, Theme) as the between-subjects factor. Where there was evidence of Sphericity we report Greenhouse-Geisser corrected p values.

Dependant Variable	F	*p*	partial η^2
Go RT	3.336	0.025	0.013
FailedStop RT	4.822	0.004	0.019
SSRT	5.139	0.003	0.033

Supplementary Table 6: Mean Go Reaction Times, FailedStop Reaction Times and SSRTs, shown separately by session number.

	Session 1 (95% CI)	Session 2 (95% CI)	Session 3 (95% CI)	Session 4 (95% CI)
Go RT	601ms (591 to 611)	614ms (602 to 626)	605ms (592 to 618)	602ms (589 to 615)
FailedStop RT	540ms (531 to 549)	556ms (544 to 568)	552ms (540 to 564)	554ms (541 to 567)
SSRT	273ms (266 to 280)	266ms (256 to 276)	259ms (248 to 270)	258ms (248 to 268)

Individual Engagement Questionnaire Data

Supplementary Figure 4 shows the scores of individual questions on the enjoyment and engagement test, calculated by averaging the long-form questionnaires from Sessions 1 and 4.

Supplementary Figure 4: Individual question scores from the subjective enjoyment and engagement questionnaire. Mean responses of VAS scores from questionnaires delivered on sessions 1 and 4, shown separately by task variant. Error bars represent 95% confidence intervals.

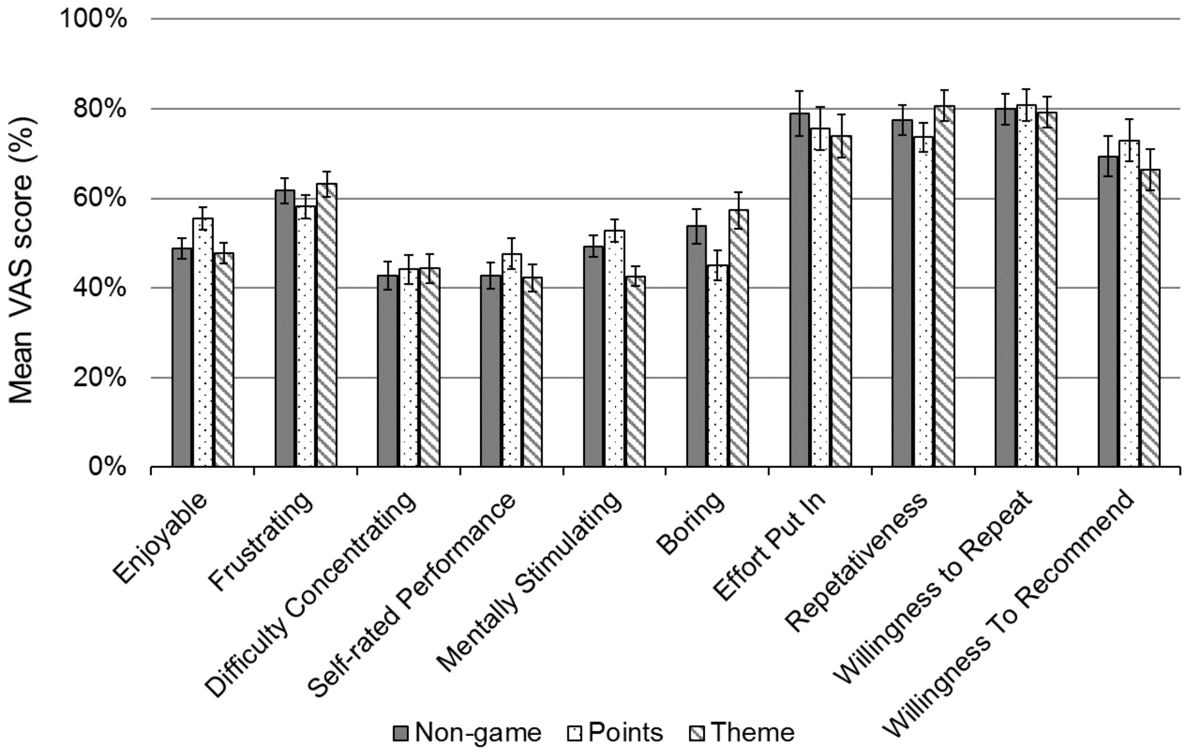

Exploratory Analyses

All Participant Attrition

Supplementary Figure 5 shows the number of participants remaining in the study at each timepoint, including all participants who signed up. We used the Kaplan Meier method to calculate the estimated survival times, and a Log-Rank test showed no evidence of a difference between the distributions (X^2 (2, N = 482) = .816, p = .67). The mean number of sessions completed in each task variant was very similar, as shown in Supplementary Table 7.

Supplementary Figure 5: Number of participants that took part each day over the ten-day period

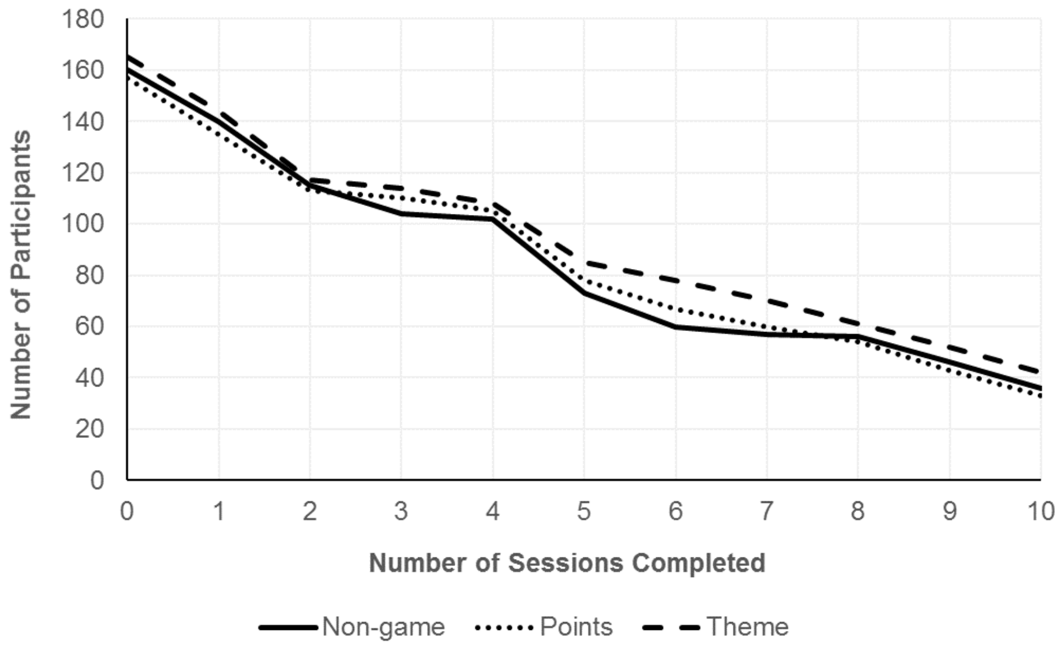

We were also interested in whether gamification would affect the number of participants who decided to stay with the study after trying one initial session. Supplementary Figure 6 shows the percentage of participants that completed a session on the first four days, divided by task variant.

Supplementary Figure 6: Percentage of participants that took part in the study each day over the first four days

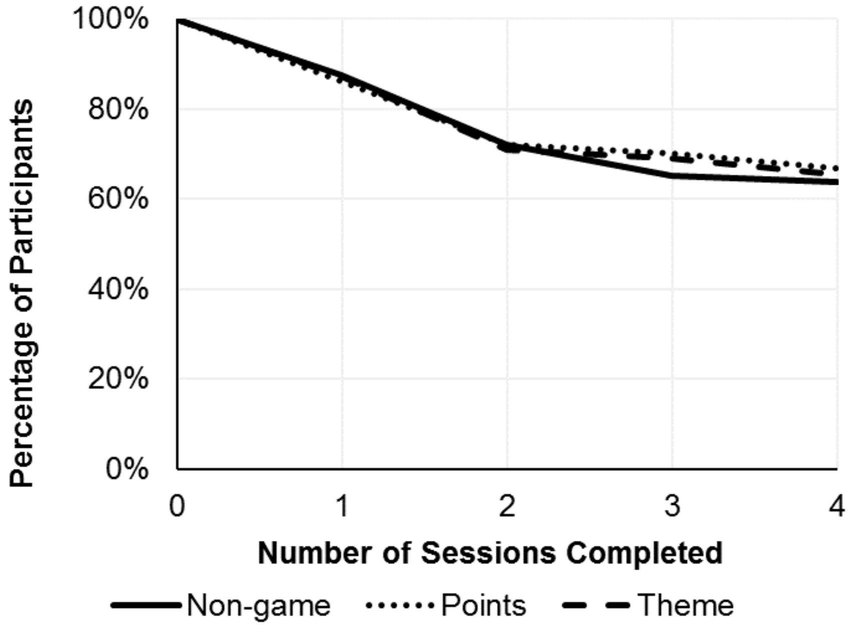

Loosely Conforming Participant Attrition

We performed an additional attrition analysis including 32 participants who only managed to complete their final compulsory test session on the fifth day of the study, rather than the fourth. As such, the maximum number of optional days they these participants could complete was 5. Collectively, we referred to the 297 participants who completed the 4 compulsory sessions within 5 days as *loosely conforming*, and the analyses below cap the maximum number of sessions completed at 9, as this is the maximum number of sessions that every participant had a chance to complete.

Supplementary Table 7: Mean number of sessions completed within 9 days, shown separately by task variant. Conforming participants are those who completed their first four sessions within four days as required. Loosely conforming participants includes conforming participants AND participants who completed their first four sessions within five days.

	All participants (95% CI)	Conforming participants (95% CI)	Loosely conforming participants (95% CI)
Non-Game	4.9 (4.4 to 5.5)	7.0 (6.6 to 7.5)	6.9 (6.5 to 7.4)
Points	5.1 (4.5 to 5.6)	7.1 (6.7 to 7.6)	7.0 (6.6 to 7.4)
Theme	5.3 (4.7 to 5.9)	7.6 (7.1 to 8.0)	7.3 (6.8 to 7.7)

Supplementary Table 7 and Supplementary Figure 7 show the attrition of these loosely conforming participants. Again, we used the Kaplan Meier method to calculate estimated survival times. A Log-rank test showed no evidence of a difference between the distributions (X^2 (2, N = 297) = 1.418, p = .49) and a one-way ANOVA of the number of sessions completed also found no evidence of a difference between task variants (F [2,296] = 0.648, p = .52, *partial* η^2 = 0.004).

We then used Bayesian t-tests to assess the mean number of sessions completed in each variant for equality. We found substantial evidence that the number of sessions completed in all variants was equal, with Points being equal to Non-Game (BF = 0.16), Points being equal to Theme (BF = 0.23) and Non-Game being equal to Theme (BF = 0.25).

Supplementary Figure 7: Percentage of loosely conforming participants that completed a session on each day of the nine-day period.

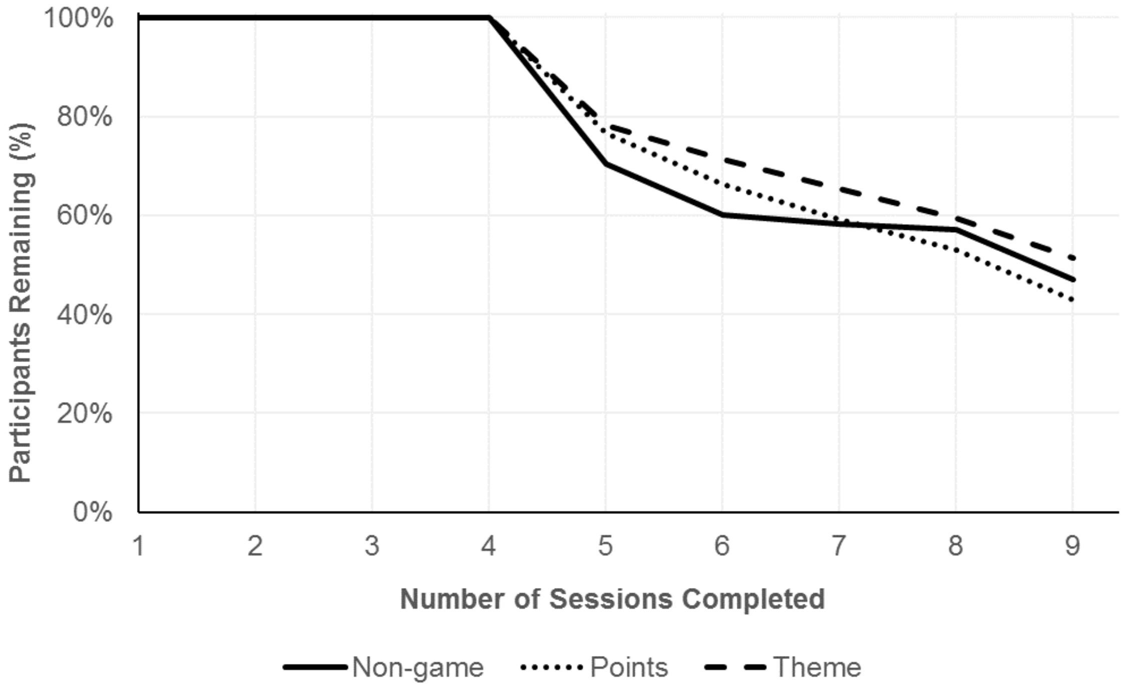

Individual differences and Attrition

To ensure that individual differences in participant perseverance between groups were not masking an effect of task variant on attrition, we used a one-way ANCOVA of mean number of sessions completed with task variant (Non-Game, Points, Theme) as the between-subjects factor and score on the perseverance questionnaire as the covariate. Again, we saw no clear evidence of an effect of task variant on the mean number of sessions completed (F [2,259] = 1.168, p = .31, *partial* η^2 = 0.009) and only weak evidence for an effect of perseverance (F [1,259] = 3.562, p = .06, *partial* η^2 = 0.013).

Previous literature has suggested that participant age, sex or their amount of video game experience can impact an individual's enjoyment of a video game, so we also ran a one-way ANCOVA of mean score with task variant (Non-Game, Points, Theme) as the between-subjects factor and age, sex and hours spent playing video games as covariates. We found no evidence for effects of the three covariates (ps > .28) and hence saw evidence of an effect of task variant on overall score (F [2,259] = 4.030, p = .02, *partial* η^2 =0.030).

References

Band, G. P. H., van der Molen, M. W., & Logan, G. D. (2003). Horse-race model simulations of the stop-signal procedure. *Acta Psychologica*, *112*(2), 105–142. https://doi.org/10.1016/S0001-6918(02)00079-3

Logan, G. D. (1994). On the ability to inhibit thought and action: A users' guide to the stop signal paradigm. In D. Dagenbach & T. H. Carr (Eds.), *Inhibitory processes in attention, memory, and language* (pp. 189–239). San Diego, CA, US: Academic Press.

Malone, T. W. (1980). What Makes Things Fun to Learn? Heuristics for Designing Instructional Computer Games. In *Proceedings of the 3rd ACM SIGSMALL Symposium and the First SIGPC Symposium on Small Systems* (pp. 162–169). New York, NY, USA: ACM. https://doi.org/10.1145/800088.802839

Malone, T. W. (1981). Toward a theory of intrinsically motivating instruction. *Cognitive Science*, *5*(4), 333–369. https://doi.org/10.1016/S0364-0213(81)80017-1

Mekler, E. D., Brühlmann, F., Opwis, K., & Tuch, A. N. (2013). Do Points, Levels and Leaderboards Harm Intrinsic Motivation?: An Empirical Analysis of Common Gamification Elements. In *Proceedings of the First International Conference on Gameful Design, Research, and Applications* (pp. 66–73). New York, NY, USA: ACM. https://doi.org/10.1145/2583008.2583017

Mekler, E. D., Brühlmann, F., Tuch, A. N., & Opwis, K. (2015). Towards understanding the effects of individual gamification elements on intrinsic motivation and performance. *Computers in Human Behavior*. https://doi.org/10.1016/j.chb.2015.08.048

Whiteside, S. P., & Lynam, D. R. (2001). The Five Factor Model and impulsivity: using a structural model of personality to understand impulsivity. *Personality and Individual Differences*, *30*(4), 669–689. https://doi.org/10.1016/S0191-8869(00)00064-7
