# Supplementary figures and images for: Attrition from Web-Based Cognitive Testing: A Repeated Measures Comparison of Gamification Techniques
Source: J Med Internet Res. 2017 Nov 22;19(11):e395. doi: 10.2196/jmir.8473 (PMC5719230; doi:10.2196/jmir.8473)

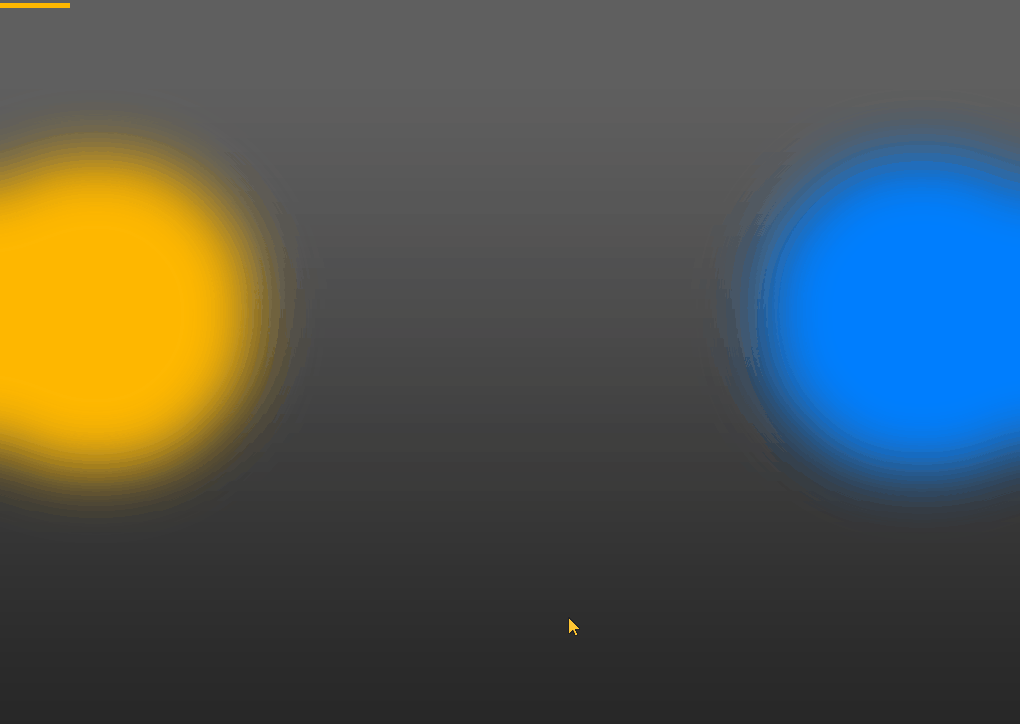

Supplement: Multimedia Appendix 2 [file jmir_v19i11e395_app2.gif]

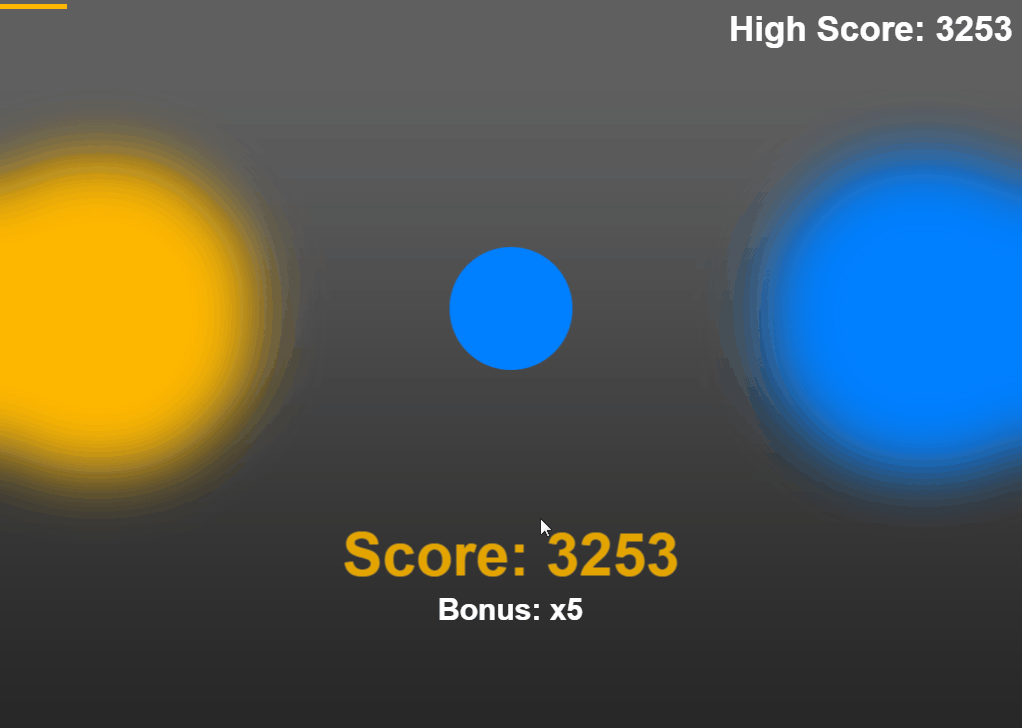

Supplement: Multimedia Appendix 3 [file jmir_v19i11e395_app3.gif]

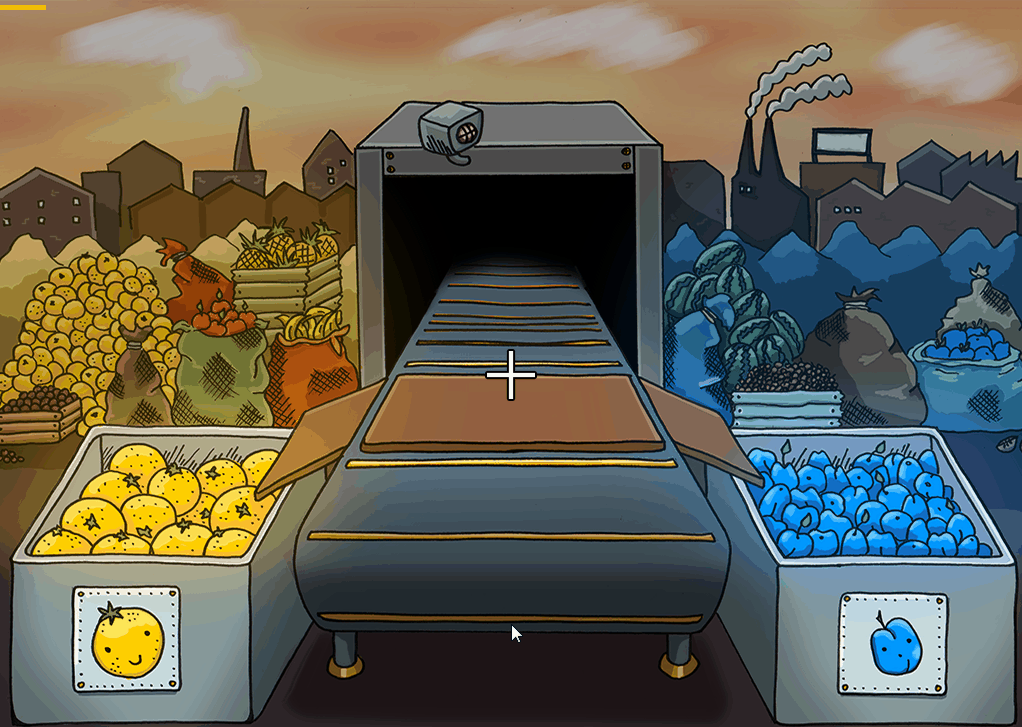

Supplement: Multimedia Appendix 4 [file jmir_v19i11e395_app4.gif]

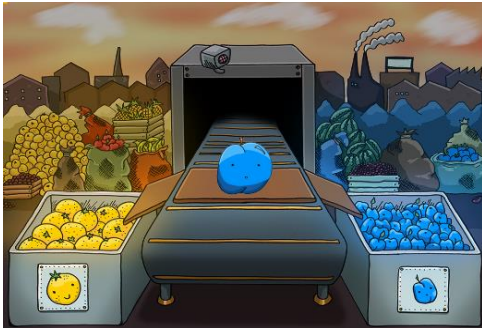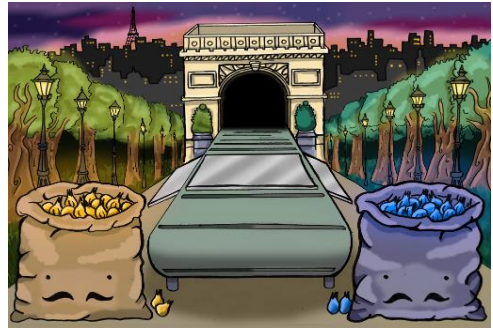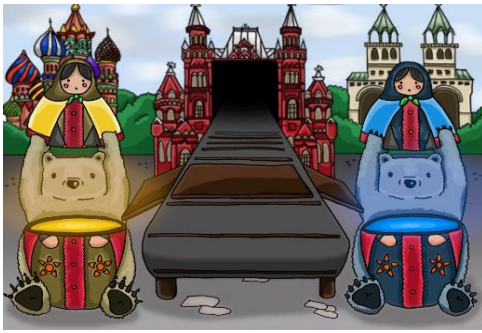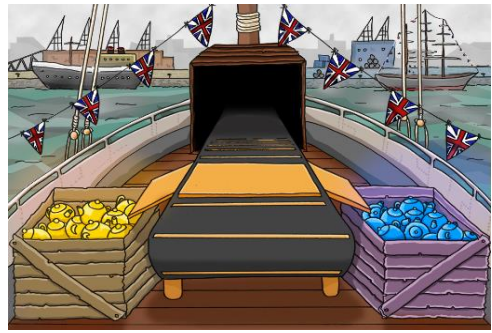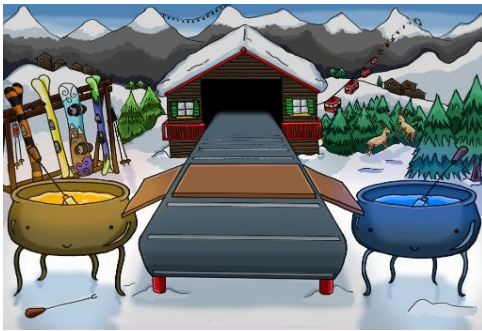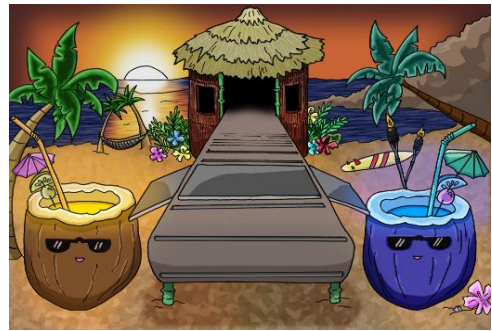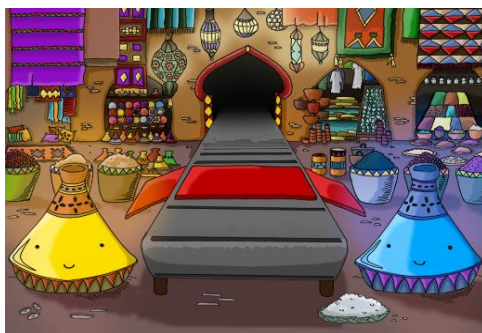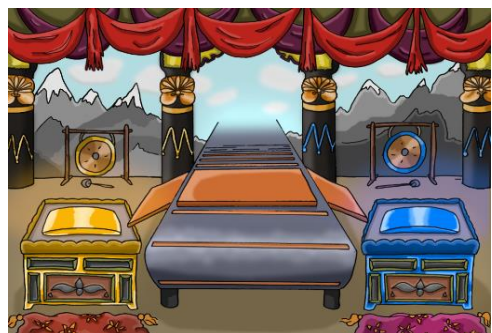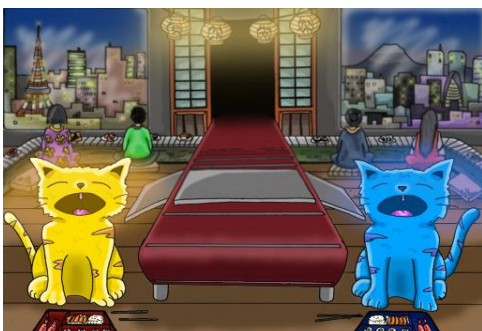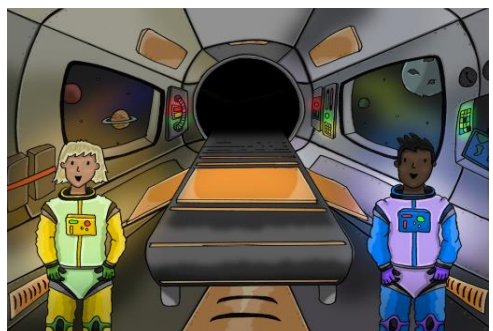

Supplement: Multimedia Appendix 5 [file jmir_v19i11e395_app5.pdf]
